# A novel epigenetic modulating agent sensitizes pancreatic cells to a chemotherapy agent

**DOI:** 10.1371/journal.pone.0199130

**Published:** 2018-06-21

**Authors:** Manjusha Thakar, Yue Hu, Michael Morreale, Lane Lerner, Wan Ying Lin, Rupashree Sen, Yi Cai, Enusha Karunasena, Maya Thakar, Soren Saggi, Harold Keer, Nita Ahuja

**Affiliations:** 1 Department of Surgical Oncology, Johns Hopkins University, Baltimore, MD, United States of America; 2 Department of Oncology, Johns Hopkins University, Baltimore, MD, United States of America; 3 Astex Pharmaceuticals, Incorporated, Pleasanton, CA, United States of America; 4 Department of Urology, Johns Hopkins University, Baltimore, MD, United States of America; University of South Alabama Mitchell Cancer Institute, UNITED STATES

## Abstract

Pancreatic ductal adenocarcinoma (PDAC) is expected to be the second leading cause of cancer mortality by 2030. PDAC remains resistant to the majority of systemic chemotherapies. In this paper, we explore if epigenetic sensitization can improve chemotherapy response in PDAC. Multiple PDAC cell lines were tested with serial concentrations of the epigenetic modulators 5-azacitidine (Aza) and guadecitabine (SGI-110). Guadecitabine was effective at inhibiting the expression of DNA Methyltransferase 1 (DNMT1) and in decreasing cell viability at nanomolar concentrations. We also report that guadecitabine has increased efficacy following a delay period or as we reference, a ‘rest period’. Sensitization with guadecitabine improved response to the chemotherapeutic agent–Irinotecan- as measured by decreased cell viability and accompanied by an increase in caspase activity. Additional studies are needed to understand the mechanism of action.

## Introduction

PDAC is currently the fourth leading cause of death due to cancer, with approximately only five percent of patients surviving five years following initial diagnosis. These abysmal rates in survival are due to a combination of PDAC’s aggressiveness, and advanced stage at primary diagnosis. Current treatment options, which include chemotherapy (i.e. Gemcitabine) as well as surgery, show limited success. A recent study shows an intensive regimen, termed FOLFIRINOX, which combines 5-Flourouracil, Leucovorin, Oxaliplatin, and Irinotecan, indicated improvements in median survival of 11.1 months compared to 6.8 months for standard systemic chemotherapy with gemcitabine [1]. However, the regimen had significant toxicity, and resistance rapidly emerges [1]. The incidence of PDAC is increasing and pancreatic cancer is expected to become the second leading cause of cancer death by 2030 [2]. Based on the aforementioned reasons, there is increasing interest to develop better treatments.

In recent years, our group has been at the forefront of understanding the role of epigenetic drugs in sensitizing cancers to chemotherapy and immunotherapy. Epigenetic changes such as DNA methylation and histone modification are able to modify overall gene expression without altering the DNA sequence. Others and we have shown that epigenetic changes are common in cancer [3–6]. In addition, use of low concentrations of epigenetic modulators can reprogram cancer cells into differentiated states [7]. In pancreas cancers, epigenetic changes occur early in carcinogenesis and may be useful for early detection in high-risk populations [3]. Through multiple collaborative efforts, pancreatic cancer sub-types have been developed based on genomics, providing better disease etiology and the identification of novel genetic disease markers [8].

Other studies have reported hyper-methylation of p16 gene promotor region in PDAC as well as early pre-neoplastic lesions such as PanIN, leading to gene silencing [9–11]. Other tumor suppressor genes such as *TP53* and *SMAD4*, are also frequently inactivated in PDAC [12–14]. DNMTs, which catalyze the DNA methylation reaction, are over-expressed in about 80% of pancreatic tumors [15–17].

5’-Azacitidine (Aza) and 5-aza-2’deoxycytidine (DAC) are FDA approved epigenetic modulators that function as DNMT inhibitors (DNMTi), and are approved for treatment in hematological malignancies such as myelodysplastic syndromes [18–21]. Clinical trials as well as preclinical data in solid tumors such as colorectal, breast, and lung cancers suggest a putative role for these epigenetic modulators as therapeutic agents, in combination with other treatment modalities such as chemotherapy or immunotherapy [22–25]. Attempts at using these therapies as single agents have been hampered by the need for prolonged treatment, as seen in hematologic malignancies as well as pharmacokinetic concerns related to their relative short half-life [26].

Given the limited half-life of the first generation DNMTi’s (Aza and DAC), there has been interest in the development of newer, more stable compounds [18]. Guadecitabine (SGI-110) is a next generation epigenetic modulator, composed of decitabine attached to deoxyguanosine, and is resistant to cytidine deaminase, resulting in a compound with a longer half-life [27–30]. In a trial composed of patients with acute myeloid leukemia, over half of the treatment naïve patients attained a complete response, with tolerable toxicity, over a range of guadecitabine doses [31]. An ongoing trial by our group in chemotherapy resistant metastatic colorectal cancer (mCRC) showed an acceptable safety profile when guadecitabine was combined with Irinotecan and a randomized Phase II clinical trial is now in progress in mCRC (NCT01896856) [32].

In this study, we examined the effect of guadecitabine on pancreas cancer cell lines, and investigated its role as a sensitizing agent for the chemotherapy drug Irinotecan. We report that guadecitabine could effectively amplify the effects of chemotherapy.

## Materials and methods

### Reagents

Pancreas cell lines were purchased from ATCC, Miapaca-2 (Cat# CRL 1420), Panc1 (Cat# CRL 1469), PL45 (Cat# CRL 2558) and Capan1 (Cat# HTB 79). MTT assay (Cat# G3580) and Caspase 3/7 (Cat# G8090) was purchased from Promega. 5-Azacitidine (Aza) was bought from Sigma (Cat# A3285). Fetal Bovine Serum (FBS) was purchased from Gemini (cat#100–106). Guadecitabine (SGI-110) was supplied by Astex Pharmaceuticals.

### Cell culture

Miapaca-2 cells were cultured in DMEM (Cat# 10-013CV) containing 10%, FBS and 2.5% horse serum (Cat# 100–508). Panc1 and PL45 cells were cultured in DMEM containing 10% FBS. Capan1 cells were cultured in IMDM (Cat# 1200–036) media containing 10% FBS. All cells were grown at 37°C in 5% CO_2_.

### Cell viability assay

For each cell line, 2000 to 5000 cells were plated per well for 24 hours prior to treatment with epigenetic modulators. Cells were seeded at a density dependent on doubling time. Cells were then treated with 5-azacitidine (Aza) (0 to 5 μM) or guadecitabine (SGI-110) (0 to 9 μM) for 1 to 5 days. Drug concentrations were calculated such that the diluents were 0.1%, and controls for each drug were treated with their respective diluent at the same concentration. Following treatment, cell viability was measured using an MTT assay according to manufacturer’s instructions (Promega). Each time point value was calculated relative to the control and expressed in terms of percent viability, and data were plotted using Graph Pad Prism.

MTT assays for measuring chemopriming by guadecitabine: Cells were treated with 0.14x10^-3^ μM of guadecitabine for three days. Cells were either plated immediately into 96 well plates (representing no rest), or reseeded into T75 flasks for 5 days, before being plated into 96 well plates (representing rested cells). Cell concentrations were 2000 cells/well; cells were allowed to adhere for 24 hours before the treatment regimen. Chemotherapy regimens consisted of Irinotecan (0–4 μM) for 5 days. Upon the completion of treatment with Irinotecan, an MTT assay was used to measure cell viability.

### Flow cytometry

Miapaca-2 and Panc-1 cells were plated into T75 flasks, and treated with guadecitabine (0, 4, and 8 μM) for three days. Once treatment was complete, the cells were fixed in 70% ethanol. Cells were stained with propidium iodide and data were acquired as described previously [33]. DIVA software was used to analyze these data.

### Western blotting

Miapaca-2 and Panc-1 cell lines were plated into T75 flasks, and treated with 0.14x10^-3^ μM of guadecitabine for 3 days. Western blots were run as previously described [34].

### Caspase 3/7 assay

A Caspase 3/7 assay was used to further evaluate the apoptotic effects of guadecitabine. Miapaca-2 and Panc1 were treated with 0.14x10^-3^ μM of guadecitabine for 3 days. Cells had either no rest, 5 days’ rest, or 10 days’ rest. Cells were then plated into 96 well plates at 2000 cells/well. Caspase activity was read as per the manufacturer’s instructions (Promega) on a luminometer. After measuring the luminescence signal, data for each concentration was divided by the respective control before plotting percent caspase activity, and the data was plotted using Graph Pad Prism.

Chemopriming activity of guadecitabine: the Caspase 3/7 assay was also used to measure apoptosis due to chemosensitization. Cells were again treated with guadecitabine at a concentration of 0.14x10^-3^ μM for 3 days. Cells were then plated into 96 well plates (representing no rest) at 2000 cell/ well, or allowed to rest for 5 days before reseeding into a 96 well plate. Cells were allowed to adhere for 24 hours before being treated with Irinotecan (0–2 μM) for five days. Caspase activity was again measured using a Promega kit, and data analysis was preformed using methods described above.

### Cytotoxicity assay

Cells were either plated 2000 cells/well (no rest) or 500 cells/ well (5 days rest). The cells were then allowed to adhere overnight and treated with 0.14x10^-3^ μM of guadecitabine for 3 days. Lactate dehydrogenase (LDH) was measured from supernatants using Thermo-Scientific Kit (88954). Data analyses were preformed according to the manufacturer’s instructions.

Chemopriming activity of guadecitabine: cells were treated with 0.14x10^-3^ μM of guadecitabine for 3 days. Cells again had either no rest or were rested for 5 days. Cells were re-plated into 96 well plates (2000 cells/ well), allowed to adhere overnight, and then treated with Irinotecan (0–4 μM) for 5 days. Cytotoxicity was measured as described above.

### Statistical analysis

Statistically significant values were calculated using parametric and non-parametric t test. Significance was defined as either (*p<0.*05*), (**p<0.*01*), or (***p<0.*001*). All experiments were replicated at least twice for reproducibility, with a minimum of technical triplicates.

## Results

### Determining the initial efficacy of different DNMTi in pancreatic cell lines

**Fig 1:**
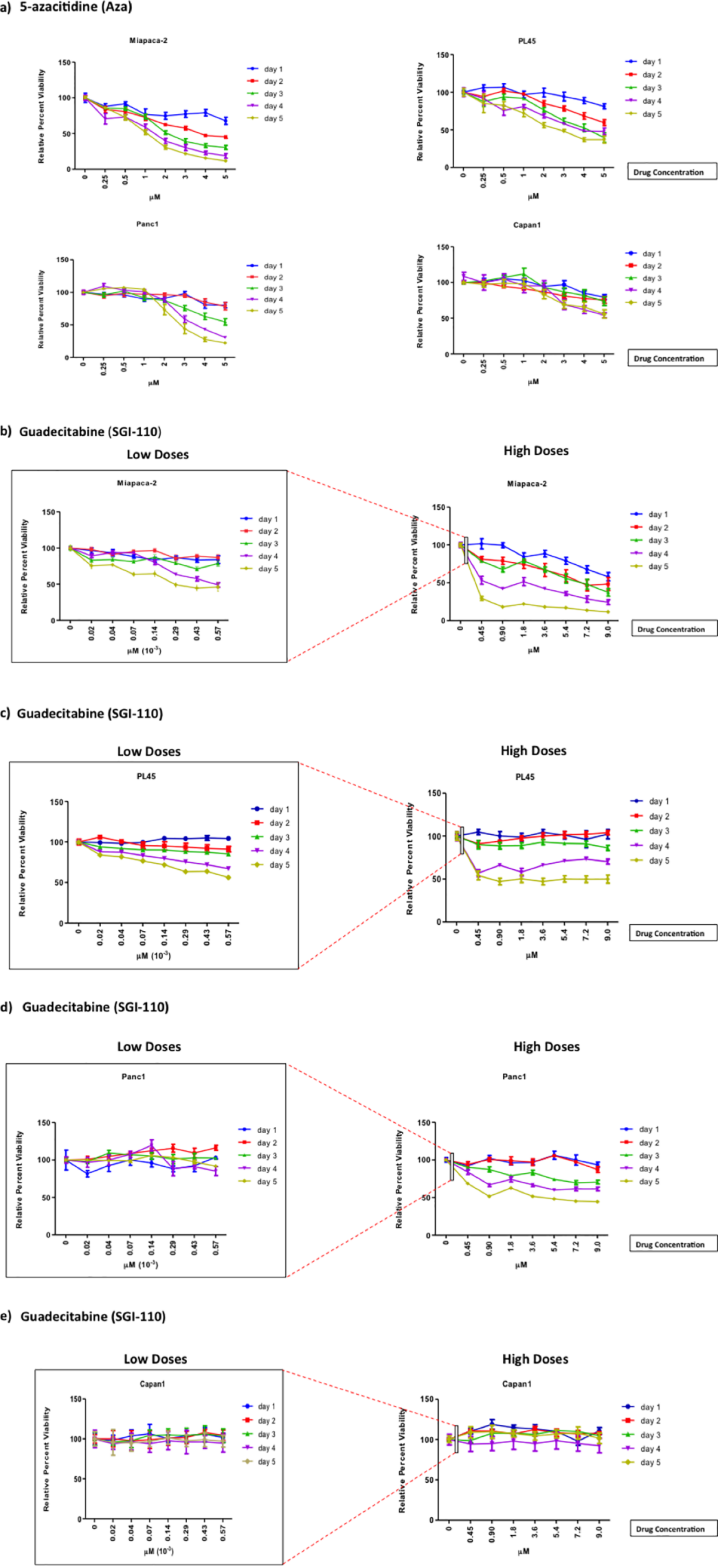
Variable response of pancreatic cell lines to epigenetic modulator *in vitro*. Cells were treated daily with an epigenetic modulator at different concentrations for up to 5 days (Fig 1A–1E). For guadecitabine, doses in nanomolar range are magnified to demonstrate the effectiveness of this modulator at low doses. At the end of the treatment, a standard MTT assay was used to determine cell viability. The percent viability was calculated by dividing each data point by its respective control and designated by percentages. Data shown represents mean ± SEM.

The cell viability of multiple pancreas cancer cell lines was measured following treatment with varying concentrations of Aza (0–5 μM) or guadecitabine (0–9 μM) (Fig 1A–1E) to determine and compare the effect of both epigenetic modulators on the pancreas cancer cell lines. For Aza, the initial decreases in viability were seen at 0.25 μM following 3 days of treatment in Miapaca-2 and PL45. For guadecitabine, differences in percent viability measures were noted at 0.02x10^-3^ μM following 3 days of treatment in the same two cell lines. In comparison, Panc1 was affected at higher doses by the epigenetic modulators (Aza: 3–5 μM and guadecitabine: 5–9 μM). However, either modulator had no effect on the percent viability in Capan1 cells.

In our progressive studies, the effect of guadecitabine was compared between a more responsive cell line (Miapaca-2), which showed viability decreases at a lower dose range (0–0.57 μM), and a less responsive cell line (Panc1), which did not show significant decreases except with higher doses of guadecitabine (0.45–9 μM).

### Impact of guadecitabine on cell cycle

As stated above, Miapaca-2 and Panc1 were the two cell lines tested. Miapaca-2 was responsive at lower doses of guadecitabine, while Panc1 was responsive at higher doses.

**Fig 2:**
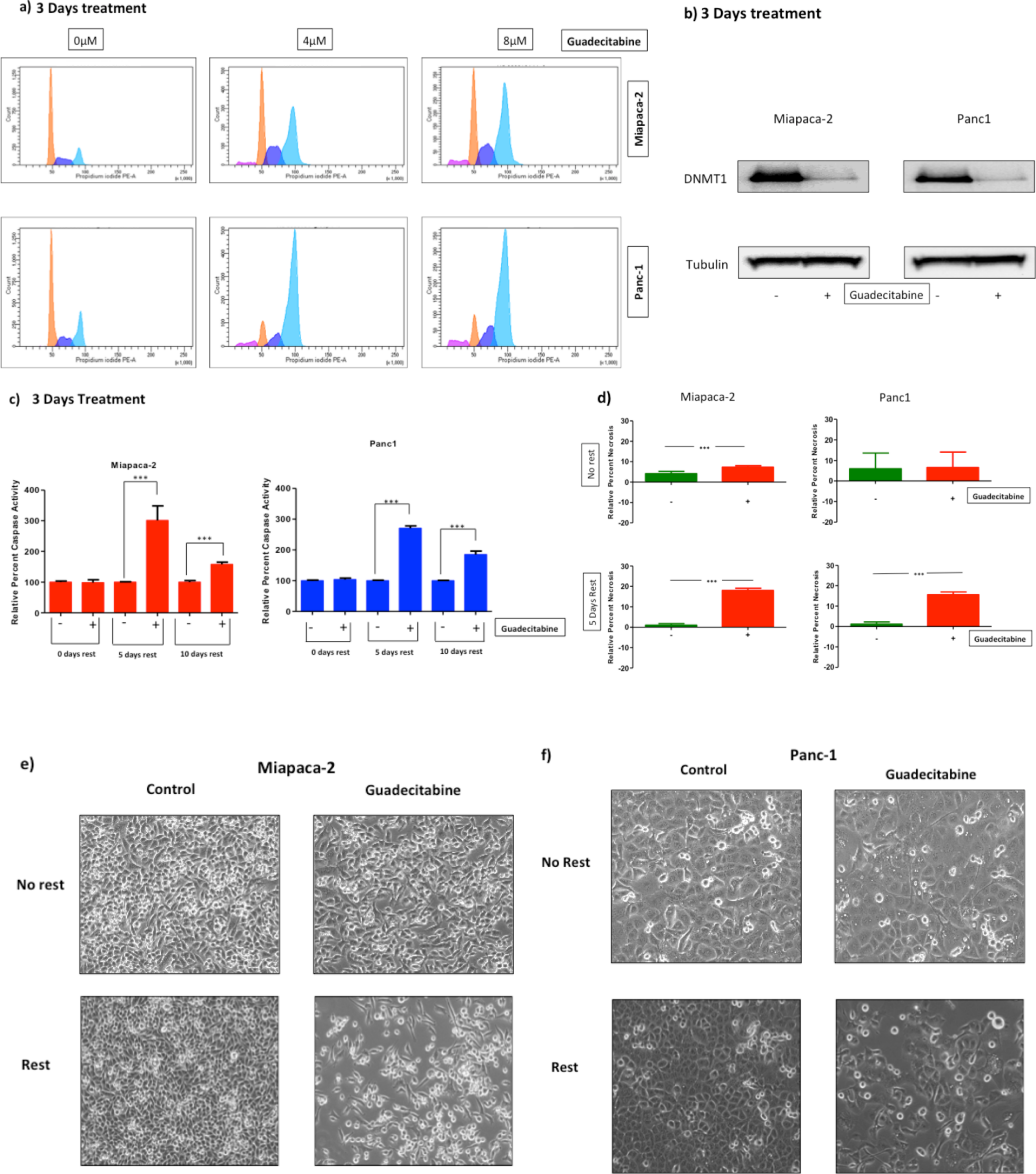
Effect of guadecitabine on DNA Methyltransferase 1, and its delayed response. Miapaca-2 and Panc1 were treated with guadecitabine (0–8 μM) for three days. The two cell lines were fixed and further stained with Propidium Iodide and analyzed using flow cytometry methods to evaluate cell cycle arrest. The pink color represents G0G1 phase, dark blue -S phase, and light blue -G2_M phase, respectively. The data was analyzed using DIVA software (a). Pancreatic cells were treated with guadecitabine at 0.14x10^-3^ μM (b-f) for 3 days. Levels of DNMT1 expression were measured via Western blotting following treatment (b). To quantify apoptotic activity, Caspase 3/7 levels were measured after 0, 5, or 10 days of rest. Each experimental value was compared with control and plotted in terms of percentages using Graph Pad Prism (c).The amount of necrosis was calculated using a LDH detection kit following treatment with no rest or rest (d). Images were taken for treated and untreated cells for Miapaca-2 and Panc1 at 10X magnification using EVOS cell imaging system (e-f).Statistical analysis was performed using a one-way ANOVA and significance measured with a Tukey’s multiple comparison test (c) or utilizing a non-parametric t test (d). Data represented shows mean ± SEM (2c-2d).

Previous work has shown that Aza is able to arrest cell cycle at the G2 phase [35]. Miapaca-2 and Panc-1 cells were treated with guadecitabine (0, 4, and 8μM) for three days in order to determine if guadecitabine acts in a similar fashion to its predecessor. Cells were stained with propidium iodide and analyzed using flow cytometry, following treatment (Fig 2A).

For Miapaca-2 cells, the percentage of cells arrested in G2_M phase was tripled (66%) in 4μM and doubled (47%) in 8μM treated groups when compared to the control (21%). Additionally, the percentage of cells arrested in G0/G1 phase increased approximately six-fold (5.3%) in the 8 μM treated group when compared to the control (0.9%). However, there was no difference between the 4 μM treated (0.5%) and the controls regarding the G0G1 phase.

For Panc1 cells, the percentage of cells arrested in G2_M phase at both concentrations was approximately doubled (71% for 4 μM and 65% for 8 μM) when compared with the control (28%). The percentage of cells arrested in G0G1 phase where roughly five-fold higher (4 μM -5.7% and 8 μM- 6.5%) than the control (1.2%) These results indicate that guadecitabine arrests both cell lines in the G2_M phase and to some extent in G0G1 phase. These observations in both cell lines support similar outcomes seen with Aza [35]; including low-dose treatment experiments. Low-doses of guadecitabine showed no demonstrable differences in cell cycle (data not shown).

### DNMT1 expression is inhibited by guadecitabine

The primary mechanism of action of guadecitabine, considered to be the inhibition of DNMT1 [36]. We tested if guadecitabine could inhibit DNMT1 at nanmolar doses (0.14x10^-3^ μM) and at which cell viability was reduced.

Miapaca-2 and Panc1 were treated with guadecitabine (0.14x10^-3^ μM) for 3 days. Following treatment, both cell lines showed reduced expression of DNMT1 (Fig 2B) via a Western Blot.

### Memory effect of guadecitabine on apoptosis

To further characterize the effect of guadecitabine at a low dose range, apoptotic activity was measured via levels of caspase after treatment with guadecitabine (0.14x10^-3^ μM) in our two-selected pancreas cancer cell lines (Fig 2C). Following 3 days of treatment, there was no observable increase in caspase activity (0 days rest). Our prior studies with 5-azacitidine in colorectal cancer and other malignancies have reflected a delayed impact due to reprogramming of cancer cells, termed a memory effect [7]. Therefore, we subsequently investigated whether caspase activity increased due to guadecitabine and after variable periods of rest (5 days vs. 10 days). The difference between control and experimental groups in caspase activity was approximately a twofold difference following 5 days of rest in control *vs* treatment (*p<0*.*001*) when compared to no difference following 0 days of rest for both cell lines. A longer period of rest (10 days) also showed an increase in caspase activity, again for Miapaca-2 and Panc1 (*p< 0*.*001*). However, the increase in caspase levels, while significant, was not as dramatic as with 5 days of rest. Therefore, in future experiments we concentrated on regimens of treatment with guadecitabine for 3 days followed by 5 days of rest.

Lactate dehydrogenase (LDH) has been shown to be a reliable indicator of necrosis [37]. A rest period of 5 days following treatment with guadecitabine (0.14x10^-3^ μM) also increased levels of necrosis in both cell lines (Fig 2D). For Miapaca-2, guadecitabine increased necrosis levels twofold with no rest (*p< 0*.*001*), but increased to eighteen-fold following 5 days rest (*p< 0*.*001*). For Panc1, guadecitabine did not significantly increase necrosis levels with no rest, but necrosis levels remarkably increased fifteen-fold following 5 days of rest (*p< 0*.*001*).

Furthermore, the effectiveness of this delay can be observed microscopically when comparing cells that were allowed to rest for 5 days *vs* 0 days following treatment with guadecitabine for 3 days (Fig 2E and 2F). The decrease in cell density observed further confirms both our caspase and necrosis assays.

### Guadecitabine sensitizes cells to the chemotherapy drug Irinotecan

Irinotecan is a topoisomerase inhibitor that has shown limited efficacy, as a single agent in PDAC. However, it has recently become an important staple in treating pancreas cancers as part of the multi-drug FOLRININOX regimen [1]. However, FOLFIRINOX is toxic and resistance to this drug gradually appears in patients [1]. Recent studies in colorectal, ovarian, and other cancers has shown an emerging role for epigenetic modulators in reversing chemoresistance [22-25]. We subsequently tested if pretreatment with DNMTi followed by Irinotecan would improve cellular response as measured by cell viability.

**Fig 3:**
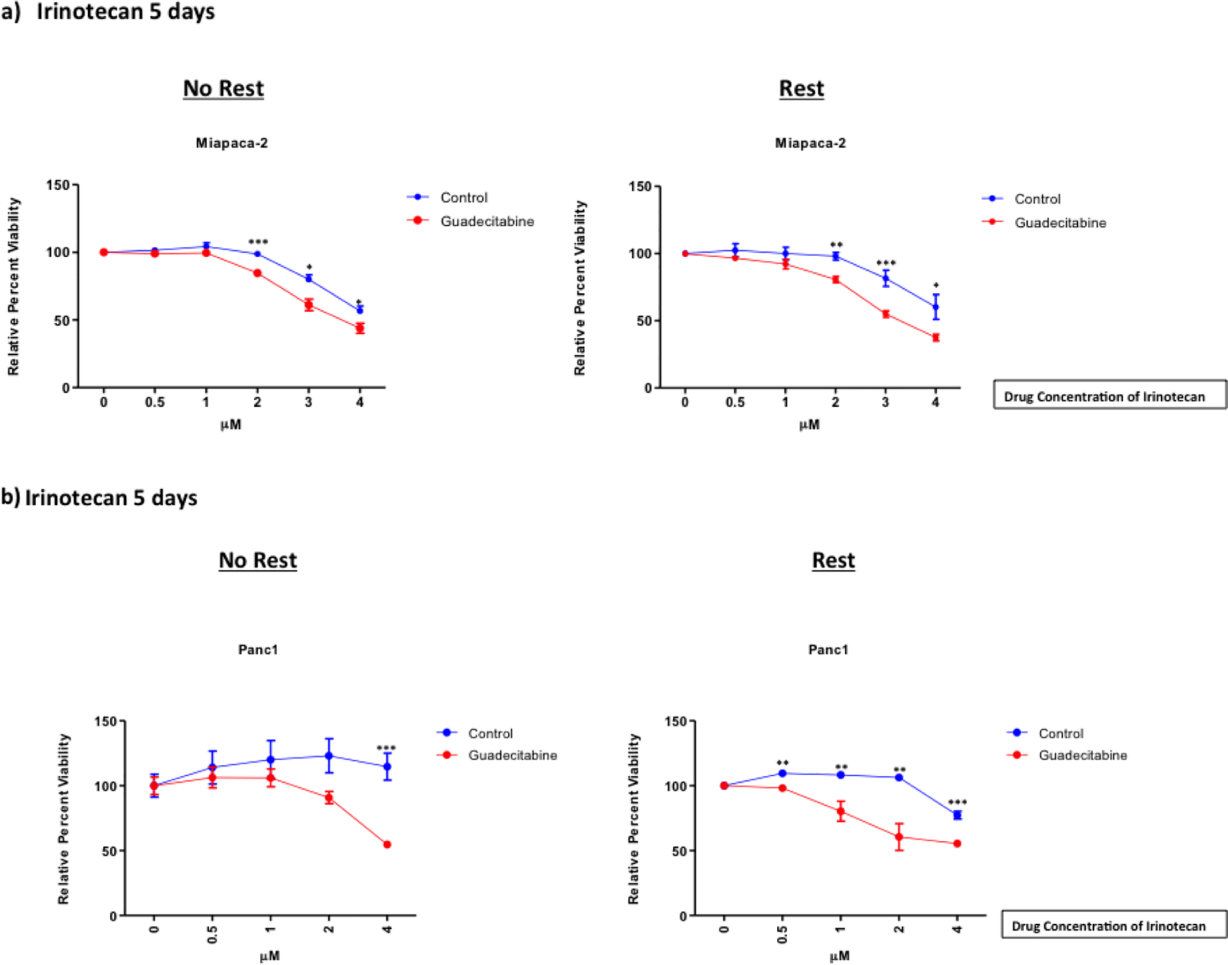
Guadecitabine sensitized PDAC cells to chemotherapy. Miapaca-2 (a) and Panc1 (b) were treated with 0.14x10^-3^ μM for 3 days. Cells were treated with varying concentrations of Irinotecan either immediately or after 5 days of rest. Cells were incubated in Irinotecan for an additional 5 days. Viability was measured using a standard MTT assay. Each data point was compared to the control respectively, and represented as percent cell viability. Statistical analysis was performed using a non-parametric t test. Data shown represents mean ± SEM.

Miapaca-2 and Panc1 cells were pretreated with guadecitabine (0.14x10^-3^ μM) for 3 days and then treated with varying concentrations of Irinotecan, after which cell viability was measured. Miapaca-2 showed enhancement in sensitivity when pretreated with guadecitabine. The improvement in sensitivity was seen after pretreatment with guadecitabine, followed by both immediate treatment and after 5 days of rest before treatment with Irinotecan (Fig 3A). However, the addition of rest did not improve Miapaca-2’s response to Irinotecan.

Pre-treatment with guadecitabine also increased Panc-1’s sensitivity to Irinotecan (Fig 3B). The addition of rest heightened the sensitivity of this cell line to Irinotecan. For 0 days rest, the first significant decrease between the control vs. the guadecitabine treated group occurred at 4 μM (*p< 0*.*001*). With 5 days rest, the first significant decrease was seen at 0.5 μM (*p< 0*.*01*), which is one eighth of the concentration needed with no rest.

### Chemosensitization with guadecitabine has no additional effect on necrosis

**Fig 4:**
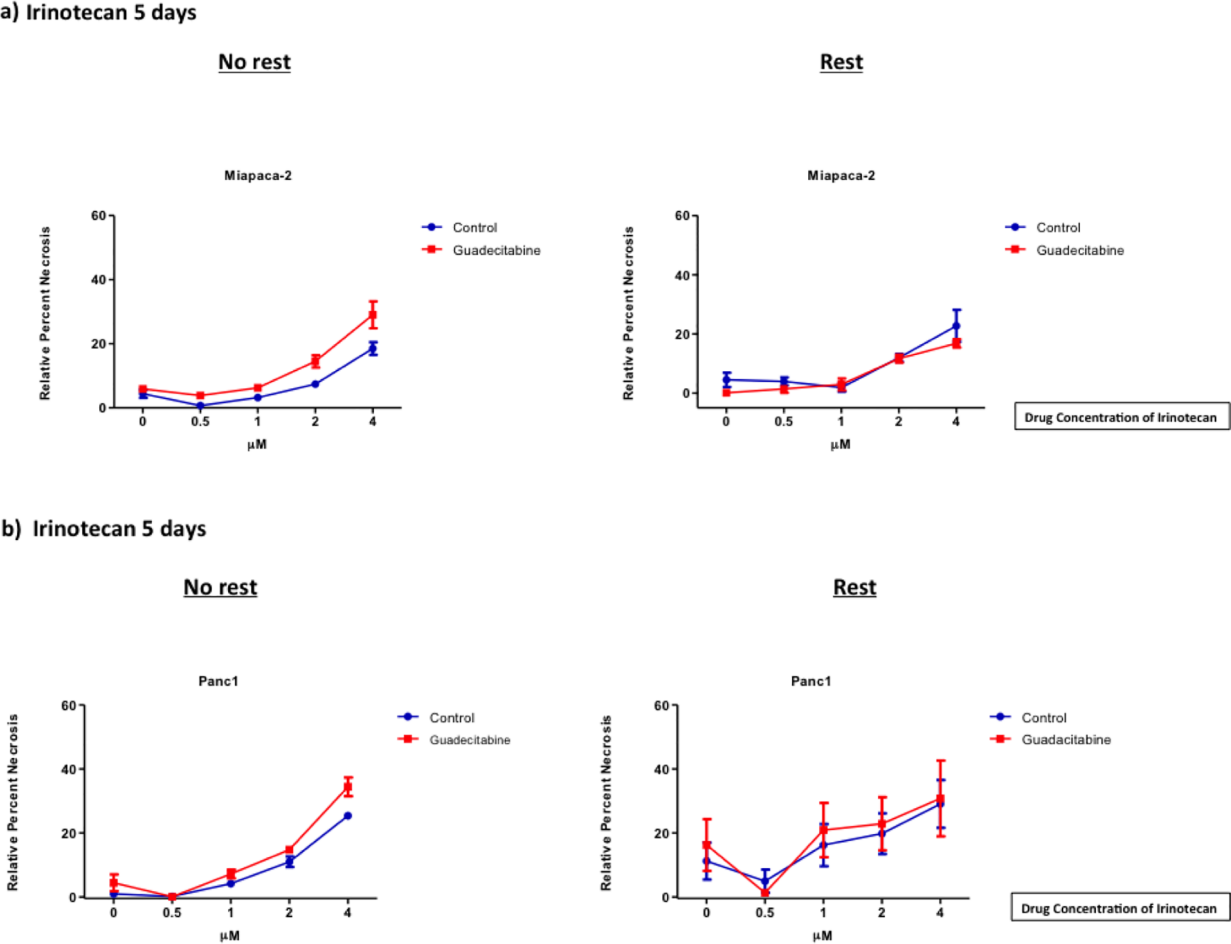
Negligible cytotoxicity in pancreatic cells due to guadecitabine. Miapaca-2 (a) and Panc1 (b) cells were treated with 0.14x10^-3^ μM of guadecitabine for 3 days and immediately treated with Irinotecan for 5 days, or allowed to rest for 5 days and then treated with Irinotecan. LDH was measured using the cytotoxicity kit mentioned previously. The data were analyzed according to manufacturer’s instructions. Analysis was done using a non-parametric t test. Data shown represents mean ± SEM.

In Miapaca-2 and Panc1, the levels of necrosis were measured in the presence of guadecitabine and Irinotecan (Fig 4A and 4B). For both lines, it was observed that the addition of guadecitabine did not increase levels of necrosis when compared to the cells that were treated with Irinotecan alone. This held true, regardless of the length of rest following treatment with guadecitabine. We next sought to determine if apoptosis also contributed to the observed decrease in viability.

### Pancreatic cells chemosensitized by a DNMTi show an increase in Caspase 3/7 activity

**Fig 5:**
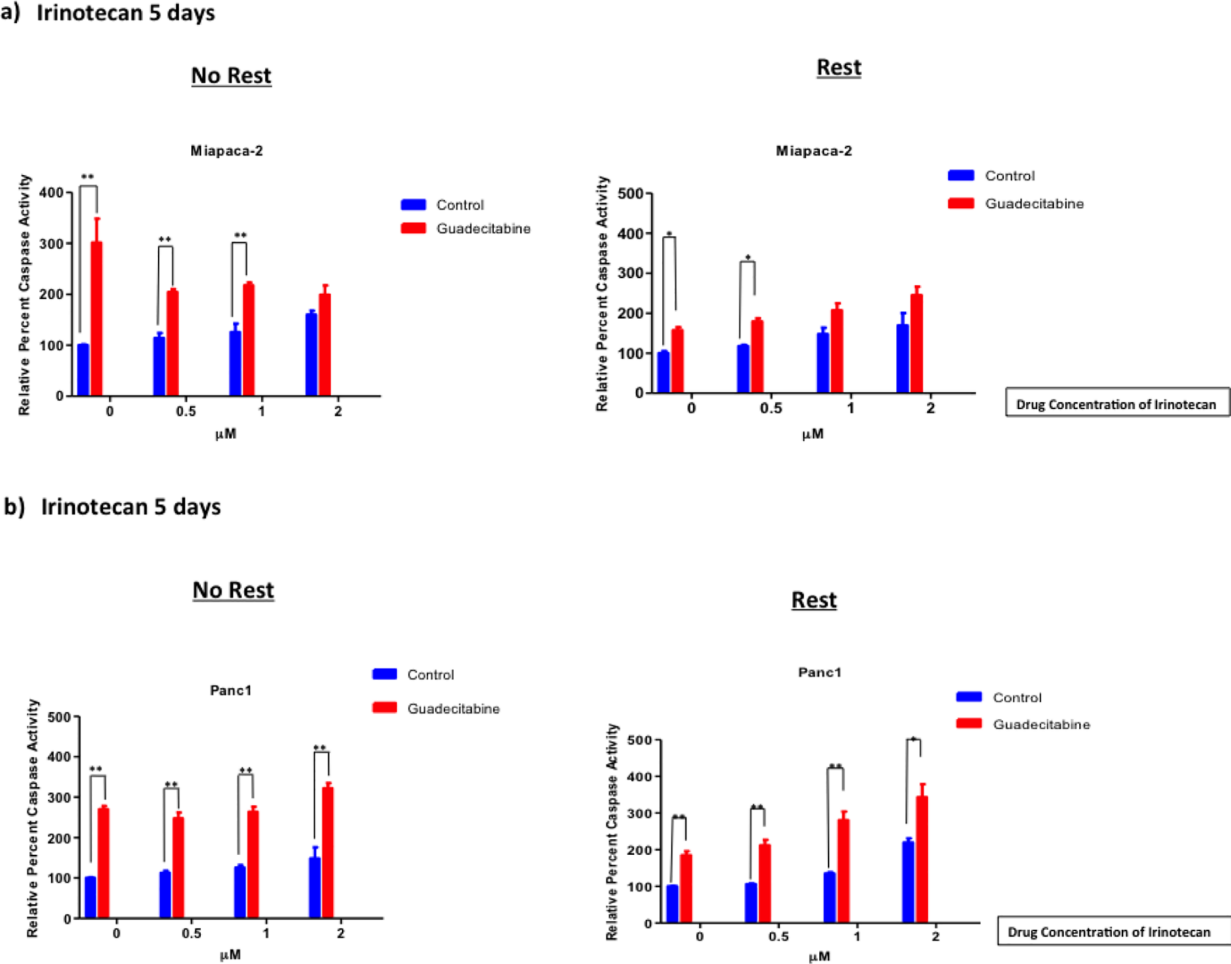
Pre-sensitized cells by guadecitabine upregulated apoptosis. Miapaca-2 (a) and Panc1 (b) cells were pretreated with 0.14x10^-3^ μM of guadecitabine for 3 days. The cells were either treated immediately or given rest for 5 days. Afterwards, the cells were treated with different concentrations of Irinotecan for 5 days. Caspase activity was measured and statistical analyses was done as mentioned earlier. A non-parametric t test was used for statistical analysis. Data shown represents mean ± SEM.

Caspase 3/7 was measured as a proxy for apoptotic activity in both cell lines, and was measured following treatment with Irinotecan alone or pretreatment with guadecitabine followed by Irinotecan as described above. In Miapaca-2 cells, caspase activity was upregulated when cells were pre-treated with guadecitabine for 3 days and then treated with Irinotecan, when compared with Irinotecan alone (Fig 5A). A period of rest prior to treatment with Irinotecan did not appreciably improve caspase activity. For both rest and no rest, pretreatment with guadecitabine and then 0.5 μM Irinotecan increased caspase activity approximately twofold when compared to 0.5 μM Irinotecan alone (*p< 0*.*05*). Correspondingly, in Panc1 the addition of guadecitabine to 0.5 μM Irinotecan significantly and equivalently increased caspase activity by around 2.5 fold when compared with Irinotecan alone, irrespective of the length of rest (*p< 0*.*01*) (Fig 5B).

## Discussion

Fundamental to DNA methylation is DNMT1 activity, which is often overexpressed in pancreatic cancer [17]. Knocking down DNMT1 noticeably inhibits cell viability in PDAC and induces apoptosis, making it an excellent target for anti-cancer therapy [38, 39]. 5-aza-2’deoxycytidine (DAC) has been tested in solid tumors including breast and gastric cancers, and PDAC; but as a monotherapy it has limited success [40–42]. DAC was used in combination either with a MEK inhibitor, Emodin, or IFN-gamma to successfully inhibit PDAC models [43–45]. Thus, additive inhibition was achieved with the use of epigenetic modulators and chemotherapy.

Epigenetic modulators are currently being explored as chemosensitizing agents in multiple tumor types [46]. Exposure to DNMTi’s leads to large B cell lymphoma cell reprogramming, sensitizing them to Doxorubicin [47]. Likewise, pretreatment with DAC sensitized pancreatic cells to Gemcitabine [48].

Additional preclinical studies show epigenetic modulators to sensitize colorectal cancer to Irinotecan [25]. Multiple clinical trials using this therapy regimen of (1) sensitizing with epigenetic modulators and (2) chemotherapy, are in progress; including a Phase 1 trial with Aza and nab-paclitaxel in solid-tumors; Aza with Gemcitabine (#NCT01167816) or Abaxane (#NCT01845805) in PDAC [49].

Guadecitabine is the next generation DNMTi. This inhibitor has the advantage of longer half-life than other available DNMTi [50]. In our preceding pre-clinical work, guadecitabine was effective at inhibiting Leiomyosarcoma in vitro and in vivo [36]. Currently, guadecitabine is being tested alone or in combination with other chemotherapies in clinical trials. A phase II trial was successful with guadecitabine in acute myeloid leukemia [31]. Following this trial, a phase III trial (#NCT02920008) was initiated and is ongoing. Likewise, a Phase I trial at Johns Hopkins was conducted using guadecitabine in combination with Irinotecan in patients diagnosed with metastatic colorectal cancers (# NCT01896856). Success from this Phase I trial has led to an ongoing international randomized Phase II trial (# NCT01896856).

In this in vitro study, guadecitabine was more effective than its predecessor Aza at decreasing cell viability in our PDAC models. At higher doses, guadecitabine decreased viability in most pancreas cell lines, CAPAN1 being the exception. At physiologically obtainable doses, guadecitabine was effective at decreasing viability in Miapaca-2 and PL45, as well as in Panc1, but to a lesser extent.

To tease out how these events transpired we investigated cell cycle arrest, previously described with Aza [35]. Therefore, we tested more and less sensitive cell lines- Miapaca-2 and Panc1. In both cell lines guadecitabine operated similar to Aza, arresting cell cycle predominantly at the G2_M phase. To further establish guadecitabine as a candidate chemopriming agent, we tested its ability to inhibit DNMT1 protein expression at low, physiological concentrations. Our results showed guadecitabine functioned effectively as a DNMTi (see, Fig 2B). Additionally, a ‘memory effect’ was observed after 5-days of rest, followed with increased Caspase 3/7 and LDH activity; similar to Aza and DAC [7] [18]. Therefore, we identified the optimal time for chemotherapy administration to be 5 days post-guadecitabine treatment, which demonstrated this memory effect.

Next, we investigated if this DNMTi would amplify the effects of Irinotecan [1]. Guadecitabine, at only nanomolar doses, significantly improved the effects of Irinotecan on decreasing cell viability, specifically with rest. This is noteworthy in Panc1, which in its aggressiveness had been largely impervious to the effects of guadecitabine or Irinotecan alone. Explicitly, rest demonstrated two additional important effects: (1) no overall increase in cell death as demonstrated by no significant changes to LDH activity (Fig 4A and 4B) and (2) initial increases in Caspase 3/7 activity were maintained during rest (Fig 5A and 5B).

We report that guadecitabine is successful at amplifying the effects of the chemotherapeutic agent Irinotecan, through chemosensitization in our PDAC model. Further studies are needed to characterize and better understand the mechanism of this combination. In comparison to current multiple-chemotherapy combination treatments, the proposed dual-combination therapy would limit the potential for adverse side effects. The success of this combination indicates opportunities for future use in clinical settings.

## Supporting information

S1 File The item called S1_File.zip included with the submission is a zip archive of PDF files containing the analyzed raw data that was used for this publication. The archive is organized by cell line, with one folder for each cell line. Within each folder, there is one file for each plot in each figure included in the text. The files are named according to the plot names in each panel of each figure, following the convention “<figure name><panel name><plot name>”. Each PDF file contains the raw data for the plot that the filename refers to.
(ZIP)

## Abbreviations

PDAC: Pancreatic ductal adenocarcinoma
Aza: 5-azacitidine
SGI-110: Guadecitabine
DAC: 5-aza-2'-deoxycytidine
DNMTs: DNA methyltranferases
LDH: Lactate dehydrogenase
IAP: Inhibitor of Apoptosis Protein
