# Correction: A novel epigenetic modulating agent sensitizes pancreatic cells to a chemotherapy agent

**DOI:** 10.1371/journal.pone.0242974

**Published:** 2020-11-20

**Authors:** Manjusha Thakar, Yue Hu, Michael Morreale, Lane Lerner, Wan Ying Lin, Rupashree Sen, Yi Cai, Enusha Karunasena, Maya Thakar, Soren Saggi, Harold Keer, Nita Ahuja

There is information missing from the Competing Interests statement. The correct Competing Interests statement is as follows: N.A. was a consultant for Cepheid, and has licensed a biomarker for early detection of pancreas cancer to Cepheid (PATENT # 10167513). N.A also has grant funding from Astex Pharmaceuticals (GRANT # 120039). N.A. has served as a consultant to Johnson and Johnson, as an advisor to Celgene, and as a member of the Scientific Advisory Council to the No Stomach for Cancer Foundation. H.K. reviewed the manuscript; he is a scientific contributor and Vice President at Astex Inc. This does not alter our adherence to PLOS ONE policies on sharing data and materials.
