# Supplementary material for: A novel epigenetic modulating agent sensitizes pancreatic cells to a chemotherapy agent
Source: PLoS One. 2018 Jun 21;13(6):e0199130. doi: 10.1371/journal.pone.0199130 (PMC6013229; doi:10.1371/journal.pone.0199130)
Supplement: S1 File — The archive is organized by cell line, with one folder for each cell line. Within each folder, there is one file for each plot in each figure included in the text. The files are named according to the plot names in each panel of each figure, following the convention “”. Each PDF file contains the raw data for the plot that the filename refers to. (ZIP) [file pone.0199130.s001.zip › Supplemental Data File/Capan 1/Figure 1a.pdf]

Figure 1a	Aza							
	0	117.363	105.711	107.226	96.706	91.208	81.787	day 1
	0.25	99.056	137.574	97.497				
	0.5	119.074	121.626	123.17				
	1	109.488	110.951	114.818				
	2	100.601	95.539	116.745				
	3	124.504	121.491	96.996				
	4	91.706	79.264	97.666				
	5	80.848	88.024	94.723				
		93.015	118.382	112.868	87.5	89.706	98.529	
		88.603	100.735	102.941				
		91.912	99.632	99.632				
		83.088	99.632	98.529				
		76.471	81.985	86.397				
		70.956	87.5	90.809				
		67.647	77.574	87.5				
		59.926	69.853	78.676				
		93.68	95.238	95.238	111.861	106.147	97.835	
		91.082	85.368	104.589				
		85.368	99.394	107.186				
		105.628	103.55	100.433				
		114.459	96.277	81.732				
		100.433	87.446	92.641				
		96.277	82.251	85.887				
		82.771	81.732	79.654				
		103.011	112.27	126.415	83.777	86.675	87.852	day2
		112.796	85.473	118.778				
		97.792	95.473	106.728				
		95.15	91.414	101.127				
		91.253	95.904	100.961				
		86.485	96.68	101.003				
		82.648	81.948	92.539				
		74.092	72.283	96.33				
		95.43039	106.9075	106.9075	99.25611	94.79277	96.70564	
		91.60468	96.70564	98.61849				
		91.60468	94.15515	96.06802				
		82.04038	79.48991	97.98087				
		75.66418	80.12753	72.47609				
		67.37513	71.83847	77.57705				
		58.44846	62.27418	64.82465				
		55.26036	55.89798	68.01275				
		99.62825	98.48287	105.8676	99.32684	100.8038	95.89069	
		93.99176	105.8977	93.50949				
		88.2347	92.30383	87.48116				
		92.2134	91.45986	90.88717				
		84.79855	91.42972	89.62122				
		73.82699	78.13725	77.44398				

85.61238	85.52195	82.86948				
83.26133	85.09997	90.46519				
93.365	104.326	101.764	97.643	92.404	110.497	Day3
89.049	100.239	98.034				
101.585	114.476	122.141				
131.412	149.328	145.784				
92.445	120.157	115.018				
107.02	108.383	105.596				
91.485	105.173	126.604				
80.91	72.54	95.119				
99.631	99.631	102.3985	88.56088	116.2362	93.54243	
98.52399	104.6125	100.738				
94.09594	107.9336	109.0406				
83.57934	92.98893	94.09594				
72.50922	76.38377	73.61623				
67.52767	69.18819	70.84871				
52.02952	54.79705	60.88561				
47.60147	53.13653	54.24354				
110.0732	107.1767	103.0939	90.48595	100.1382	89.03204	
105.2574	114.7772	106.5811				
103.7861	115.0551	95.54777				
96.17759	104.705	107.9515				
93.52359	101.1726	93.86665				
84.29269	86.78925	82.368				
85.8111	83.77253	75.6438				
86.52042	93.97865	80.69085				
99.352	116.657	88.523	96.247	99.764	99.458	day 4
125.572	121.909	135.547				
125.841	130.126	127.089				
115.297	126.803	127.589				
107.503	137.021	104.291				
84.397	99.897	97.166				
83.236	86.616	80.431				
65.447	72.044	62.366				
103.2364	102.7448	106.186	153.134	144.5309	144.2851	
106.4318	105.9402	125.3585				
102.7448	107.6608	104.2196				
94.38754	91.68374	96.84556				
78.16469	75.21507	83.5723				
63.66243	64.89144	66.36624				
52.84719	50.63499	52.84719				
42.27776	46.70217	47.43958				
82.86755	48.35909	107.2036	114.219	122.069	125.2819	
53.73021	56.6427	67.29089				
84.64636	62.57206	99.66035				
28.9841	88.36665	88.90254				

82.98236	72.17928	115.6564
36.14014	50.53357	60.06545
41.82572	54.31077	55.98229
57.03371	44.83039	48.70745

95.60327	104.8057	96.01227	98.46626	104.8057	100.3067	day5
85.17382	91.10429	91.30879				
77.19836	84.15133	92.12679				
69.8364	74.53988	80.26585				
56.95296	60.42945	62.47444				
42.84254	48.15951	57.15746				
39.36605	41.20654	41.61554				
28.32311	35.88957	35.68507				

93.74872	115.2737	90.97758	111.495	106.863	102.352	86.418	98.596	94.275
102.1567	81.99965	121.7353	103.621	94.516	102.893			
100.9915	111.9126	132.1443	95.894	102.253	92.306			
96.96607	122.5194	110.0023	110.897	112.635	106.094			
85.48541	95.86942	105.676	88.939	91.306	104.085			
69.97569	74.72516	96.76685	77.791	77.833	77.402			
75.47908	73.69352	73.76582	78.424	81.051	88.144			
65.59125	70.27049	70.61489	69.277	67.878	59.448			
