# Supplementary material for: A novel epigenetic modulating agent sensitizes pancreatic cells to a chemotherapy agent
Source: PLoS One. 2018 Jun 21;13(6):e0199130. doi: 10.1371/journal.pone.0199130 (PMC6013229; doi:10.1371/journal.pone.0199130)
Supplement: S1 File — The archive is organized by cell line, with one folder for each cell line. Within each folder, there is one file for each plot in each figure included in the text. The files are named according to the plot names in each panel of each figure, following the convention “”. Each PDF file contains the raw data for the plot that the filename refers to. (ZIP) [file pone.0199130.s001.zip › Supplemental Data File/Capan 1/Figure 1e high dose.pdf]

Figure 1e	SGI	HIGH dose						
	0	104.8801	104.9942	120.8907	97.51855	94.96541	76.75092	day 1
	0.45	129.0491	122.6218	114.063				
	0.9	128.1058	147.2382	147.072				
	1.8	122.9629	136.7363	114.4274				
	3.6	128.4798	138.7807	86.61446				
	5.4	114.0563	103.358	112.1037				
	7.2	96.76778	81.29007	66.11707				
	9	128.2246	133.641	107.0272				
		107.2626	105.0279	99.44134	99.44134	90.50279	98.32402	
		115.0838	107.2626	100.5587				
		113.9665	106.1452	102.7933				
		111.7318	108.3799	111.7318				
		117.3184	116.2011	102.7933				
		110.6145	110.6145	107.2626				
		111.7318	101.676	108.3799				
		105.0279	98.32402	98.32402				
		88.7522	91.3884	115.6415	95.07909	102.9877	106.1511	
		100.3515	104.5694	99.82425				
		108.2601	104.5694	111.4236				
		109.8418	108.2601	110.8963				
		110.8963	108.2601	111.9508				
		109.8418	115.6415	109.8418				
		105.6239	108.7873	100.8787				
		107.7329	104.0422	114.0598				
		115.7895	111.6147	97.14189	94.86268	89.18218	91.40899	day2
		113.2623	103.0097	112.0816				
		110.2901	109.3688	93.74578				
		97.01308	104.264	85.57841				
		98.85097	115.0956	115.6103				
		99.61826	99.86726	89.83263				
		93.94455	96.31007	95.28661				
		108.3649	106.8952	105.6592				
		99.28058	93.11408	101.1305	106.0637	101.1305	99.28058	
		122.7133	116.5468	109.7636				
		123.9466	117.1634	108.5303				
		110.9969	128.8798	115.3135				
		122.7133	133.8129	114.6968				
		124.5632	135.6629	114.6968				
		127.6465	125.7965	114.6968				
		104.8304	122.0966	117.7801				
		103.595	106.9898	106.2087	94.04166	96.1446	93.02023	
		107.7709	107.861	103.1444				
		111.1656	110.9854	110.6249				
		109.1829	106.8095	103.3247				
		99.83978	103.6251	107.9812				
		106.8696	102.3333	102.3032				
		102.4234	105.4276	102.4835				
		97.46645	101.3118	105.7581				
		106.8911	111.0321	114.497	85.19525	85.75248	96.63203	day 3
		97.90375	90.78791	86.36228				

114.2926	114.655	112.7765				
127.883	123.2759	107.9277				
109.7817	91.06253	102.9444				
108.13	104.0196	107.3506				
109.5168	111.5477	111.3201				
91.0198	102.9823	103.847				
97.46525	99.91824	105.3148	98.93704	98.44644	99.91824	
90.59689	103.843	94.03107				
107.7678	103.843	95.01227				
103.3524	95.01227	100.4088				
112.6738	105.8054	105.3148				
122.4857	124.4481	115.1267				
113.1644	117.5797	111.202				
116.5985	113.1644	112.1832				
108.8634	107.8124	102.1517	94.7378	90.35473	96.07992	
103.7473	113.3345	108.3015				
106.0311	110.1379	105.7742				
110.9345	101.9097	102.1302				
116.3267	110.0501	104.52				
110.8086	103.8691	104.8506				
108.948	107.9704	98.79456				
104.0672	102.8917	96.25541				
106.6163	110.7107	100.3515	94.26891	83.64854	104.4041	day 4
127.606	103.5533	124.3575				
118.9983	121.4966	108.505				
128.2852	134.0689	130.299				
121.5112	110.5663	115.9823				
127.9509	124.2552	123.4845				
112.2599	110.9643	122.3498				
116.7343	112.1158	119.5201				
105.3179	97.64095	90.44382	102.1991	106.5174	97.88084	
111.3155	100.9996	101.7193				
104.5982	112.9948	104.5982				
99.80008	107.9568	97.88084				
112.2751	106.9972	104.1184				
111.0756	113.7145	111.3155				
114.914	109.8761	112.9948				
101.7193	101.7193	105.5578				
42.28994	59.28576	62.23507	145.3964	153.0229	137.77	
55.38128	55.01321	69.48048				
49.97485	66.86072	68.05091				
58.12611	65.95677	59.85194				
58.50244	81.67068	43.39446				
55.58181	49.80389	70.47514				
53.33616	61.70978	58.28737				
49.14251	52.83718	73.72075				
99.591	100.818	96.31902	103.681	111.0429	107.1575	day5
79.14111	90.59305	79.7546				
80.3681	86.70757	88.13906				
88.75256	93.04704	90.79755				
89.57055	85.88957	83.84458				

95.91002	90.59305	79.7546						
78.93661	78.52761	78.11861						
73.61963	72.18814	85.07157						
52.50118	120.7727	116.0963	90.03278	97.26718	95.33723	114.2287	98.83181	104.3023
122.3582	119.5788	138.8377	124.4111	113.8093	112.2025			
133.0938	145.2716	141.2877	103.3297	107.7187	100.0006			
117.3128	116.8547	105.3417	120.1889	120.1928	116.6588			
117.7574	119.9523	103.2292	123.794	111.8724	104.9921			
104.6653	117.3393	114.2101	118.3704	123.5876	118.481			
120.2745	117.0108	128.3661	133.7926	121.3451	116.3114			
120.7081	111.4992	118.5923	106.0629	107.0701	119.4894			
