# Supplementary material for: A novel epigenetic modulating agent sensitizes pancreatic cells to a chemotherapy agent
Source: PLoS One. 2018 Jun 21;13(6):e0199130. doi: 10.1371/journal.pone.0199130 (PMC6013229; doi:10.1371/journal.pone.0199130)
Supplement: S1 File — The archive is organized by cell line, with one folder for each cell line. Within each folder, there is one file for each plot in each figure included in the text. The files are named according to the plot names in each panel of each figure, following the convention “”. Each PDF file contains the raw data for the plot that the filename refers to. (ZIP) [file pone.0199130.s001.zip › Supplemental Data File/Capan 1/Figure 1e Low dose.pdf]

Figure 1e	SGI	Low dose												
	0	101.6	101.32	89.57	95.04	87.93	89.026	116.35	111.2	107	98.3	100	102.4	day 1
	0	102.4	98.042	93.67	96.68	95.04	94.217							
	0	91.21	96.949	105.4	96.13	98.32	98.042							
	0	101.9	105.42	96.95	85.75	97.22	98.042							
	0	101.3	104.6	98.32	102.7	96.68	100.5							
	0	100.5	96.949	103.8	99.14	100.5	101.05							
	0	102.7	101.87	97.77	95.04	98.04	102.96							
	1	93.67	100.5	100.8	93.94	93.12	109.52							
		95.86	91.642	104.3	97.48	103.7	103.98	91.966	97.48	105	110	100	98.78	
		89.37	106.25	95.21	113.1	99.43	94.239							
		104.6	113.07	108.8	111.8	110.5	111.44							
		103.3	111.44	121.8	119.2	123.8	114.04							
		97.48	102.68	92.62	107.5	93.27	105.6							
		103.7	106.25	101.1	120.2	103.7	110.47							
		117.3	107.55	115.7	117	108.8	111.12							
		103	107.87	105.9	104.6	103.7	102.68							
		102.7	100.76	103.2	101.4	95.97	104.68	106.64	100.5	97.3	91.6	93	101.9	day2
		112.9	105.11	102.5	107.9	108.4	105.99							
		109.2	112.08	106.6	103.6	105.6	105.99							
		114	105.11	105.8	100.3	106.9	108.38							
		114.9	106.64	105.8	109.2	104.7	107.73							
		110.8	105.99	108.8	105.8	105.1	108.16							
		111.4	112.51	111.9	101.6	110.3	108.38							
		109	112.3	106.9	101.4	97.72	116.87							
		95.46	92.225	91.87	100.1	95.81	105.14	113.76	103	103	98	98	104.1	
		96.53	84.33	83.25	88.64	97.25	109.09							
		100.5	81.1	76.08	80.38	91.51	101.2							
		96.17	83.254	80.02	83.97	100.5	103.35							
		98.68	90.789	87.56	90.07	92.58	104.79							
		108	93.301	91.87	89.35	95.1	98.684							
		111.2	103.35	102.3	107.3	108	108.73							
		105.5	100.48	98.33	95.46	104.4	108.01							
		104.6	101.58	94.67	94.85	88.64	90.769	107.43	103.5	105	99.5	100	110.1	day3
		96.62	88.643	93.43	101.6	93.61	99.454							
		99.63	82.44	93.43	96.97	73.05	97.504							
		102.3	103.18	102.1	99.45	95.73	100.16							
		101.6	103.18	99.45	103.2	101.6	105.3							
		103.4	97.504	93.96	98.75	96.97	102.47							
		100.2	103.71	103.9	98.04	102.1	104.77							
		108.1	105.83	95.73	93.07	100.2	100.87							

92.53	97.581	104.7	91.69	91.69	96.318	114.2	111.7	111	83.1	97	108.7	
99.68	99.684	94.64	95.06	99.26	96.95							
104.5	103.05	92.95	97.79	96.95	111.89							
101.4	97.791	100.5	94.22	104.1	104.73							
109.4	102.63	99.9	93.58	95.27	104.31							
106.6	97.581	100.7	98.21	97.37	110.84							
102.6	105.58	93.16	88.32	100.3	108.1							
116.1	103.26	104.1	90.22	91.69	103.47							
80.85	95.085	91.7	97.46	106.6	96.78	99.153	104.2	103	110	104	111.4	
95.09	92.034	89.66	95.09	99.83	100.17							
99.15	100.85	101.2	99.15	104.6	103.22							
110.3	107.63	111.4	111	120.2	113.73							
102.2	105.93	107.3	110.3	113.7	128.98							
103.2	99.831	111	119.2	117.1	126.27							
105.3	107.63	113.4	128.3	117.5	126.61							
98.81	110	104.6	114.1	106.3	124.24							
99.36	95.808	95.81	98.65	92.25	97.763	103.81	106.7	105	98.5	102	103.6	day4
103.3	98.297	99.54	92.96	91.19	96.341							
104	98.474	103.5	102.4	93.14	99.008							
104	106.65	104	102.9	99.9	100.96							
106.5	109.5	104	104.9	95.81	105.05							
103.8	104.34	110.6	109	100.1	99.363							
103.8	99.185	107.4	101	98.83	104.52							
104.3	95.097	109.9	91.19	102.7	118.92							
85.66	81.963	85.5	93.87	97.9	95.803	124.77	88.72	101	122	122	100.8	
82.12	85.343	81.64	89.04	94.03	99.987							
81.96	77.779	83.73	89.04	91.62	100.15							
84.22	73.113	73.76	88.08	98.22	93.872							
85.34	76.492	82.29	91.3	90.81	98.86							
85.18	82.285	83.57	89.69	95.48	101.44							
84.86	84.86	86.15	91.46	93.55	98.699							
86.15	80.515	84.22	88.56	95	99.021							
85.45	92.75	97.4	106.4	99.39	105.04	114.33	121.3	103	79.8	97	97.4	
91.09	101.05	97.07	105	90.76	90.758							
88.43	106.36	110	100.4	89.76	117.99							
93.08	91.422	92.42	79.47	99.06	109.02							
96.74	99.723	100.1	96.74	89.43	124.96							
93.75	101.72	102.7	51.91	96.07	119.98							
102	107.03	95.08	82.46	89.1	102.71							
102	100.72	98.06	80.13	85.45	79.801							
134.2	97.316	97.47	94.99	96.7	92.826	100.41	98.71	101	95.8	95	95.77	day5
150.3	107.23	101.3	98.4	100.7	96.387							

122.9	101.34	101.5	101.8	99.02	99.794
105.5	99.484	104.7	102	102.3	101.03
104	102.43	104.7	106.1	104.4	106.92
99.95	100.41	100.9	105.2	100.4	107.54
110.3	108	108.2	106.1	104	103.51
102	102.89	104.9	97.47	97.01	96.697

96.55	92.071	93.67	95.06	96.12	99.644	105.4	101.5	105	103	104	107.1
90.26	86.844	85.99	91.75	97.3	102.2						
90.15	74.044	86.1	90.9	94.63	100.39						
90.9	86.524	83.75	92.6	97.4	102.63						
89.08	87.804	88.98	95.48	97.72	104.66						
96.23	92.284	88.12	93.14	93.46	100.18						
93.03	88.978	92.92	95.38	99.32	103.16						
92.28	91.218	87.27	96.87	98.36	105.08						

107.8	90.716	91.87	95.06	98.82	112.42	95.346	84.35	93.9	102	108	119.7
92.74	81.746	82.33	74.8	83.48	98.529						
91.01	94.189	86.95	85.8	90.14	99.108						
93.03	90.716	97.08	93.32	98.53	101.13						
111.6	95.635	106.3	100.8	99.4	119.36						
95.93	91.874	86.38	97.08	95.35	117.92						
82.04	108.66	86.67	93.32	92.74	107.5						
91.58	85.797	95.06	88.11	100.8	115.31						
