# Supplementary material for: A novel epigenetic modulating agent sensitizes pancreatic cells to a chemotherapy agent
Source: PLoS One. 2018 Jun 21;13(6):e0199130. doi: 10.1371/journal.pone.0199130 (PMC6013229; doi:10.1371/journal.pone.0199130)
Supplement: S1 File — The archive is organized by cell line, with one folder for each cell line. Within each folder, there is one file for each plot in each figure included in the text. The files are named according to the plot names in each panel of each figure, following the convention “”. Each PDF file contains the raw data for the plot that the filename refers to. (ZIP) [file pone.0199130.s001.zip › Supplemental Data File/Miapaca-2/Figure 1 b High dose.pdf]

Figure 1b	SGI	High dose						
	0	108	89	112	96	105	90	day1
	0.45	83	110	125				
	0.9	102	85	102				
	1.8	73	67	98				
	3.6	71	81	100				
	5.4	61	71	89				
	7.2	55	55	65				
	9	46	39	60				
		92	101	97	96	101	112	
		83	106	102				
		103	97	109				
		89	96	83				
		93	96	90				
		79	90	84				
		81	73	78				
		61	73	70				
		92	106	100	107	96	100	day2
		76	79	68				
		64	72	71				
		66	56	68				
		49	49	51				
		43	44	38				
		28	36	28				
		29	28	31				
		89	109	113	92	98	99	
		90	86	89				
		93	87	87				
		90	82	84				
		92	67	93				
		77	79	75				
		58	71	59				
		66	70	66				
		97	103	108	95	95	102	day3
		78	76	76				
		78	67	56				
		83	88	74				
		78	67	56				
		40	43	43				
		34	38	30				
		26	27	27				

95	100	98	94	107	106	
82	78	81				
61	71	72				
80	77	72				
61	71	72				
66	72	70				
62	59	65				
47	53	44				
113	85	106	98	93	105	day4
72	47	66				
41	46	44				
60	57	66				
41	46	44				
41	41	42				
40	41	32				
38	29	24				
100	125	110	88	85	92	
39	44	52				
38	37	48				
30	43	53				
38	37	48				
31	30	30				
17	20	21				
18	18	19				
110	94	90	116	85	104	day5
42	31	25				
22	19	18				
25	20	20				
22	19	18				
18	17	14				
16	15	13				
14	14	14				
100	108	101	92	98	102	
29	26	24				
18	15	17				
25	19	23				
18	15	17				
18	22	13				
15	11	12				
10	8	9				
