# Supplementary material for: A novel epigenetic modulating agent sensitizes pancreatic cells to a chemotherapy agent
Source: PLoS One. 2018 Jun 21;13(6):e0199130. doi: 10.1371/journal.pone.0199130 (PMC6013229; doi:10.1371/journal.pone.0199130)
Supplement: S1 File — The archive is organized by cell line, with one folder for each cell line. Within each folder, there is one file for each plot in each figure included in the text. The files are named according to the plot names in each panel of each figure, following the convention “”. Each PDF file contains the raw data for the plot that the filename refers to. (ZIP) [file pone.0199130.s001.zip › Supplemental Data File/Miapaca-2/Figure 1a.pdf]

Figure 1a	AZA							
	0	83	90	91	163	79	94	day1
	0.25	93	77	76				
	0.5	97	83	84				
	1	70	55	62				
	2	59	77	63				
	3	80	63	66				
	4	86	66	65				
	5	79	49	58				
		96	107	117	90	93	97	
		91	94	97				
		95	91	100				
		76	98	100				
		75	88	87				
		76	92	87				
		83	83	92				
		67	79	75				
		78	98	118	106	103	96	day2
		84	84	64				
		77	80	65				
		70	65	58				
		63	62	63				
		68	59	55				
		54	49	45				
		54	49	45				
		99	91	105	101	106	98	
		85	95	90				
		87	88	88				
		78	79	86				
		61	61	65				
		52	52	60				
		44	46	46				
		39	41	45				
		96	101	99	107	104	94	day3
		86	84	92				
		83	84	93				
		82	73	85				
		53	60	57				
		49	48	44				
		39	40	39				
		39	37	34				

96	102	93	104	109	96	
86	80	86				
78	80	91				
70	65	67				
46	47	45				
32	33	28				
27	25	29				
24	24	25				
105	122	100	104	90	79	day4
42	80	54				
84	81	61				
54	58	77				
38	51	50				
36	41	41				
30	27	29				
24	24	29				
85	100	116	101	86	113	
85	84	79				
67	75	71				
46	55	64				
30	33	36				
18	22	24				
17	18	17				
9	13	14				
104	99	110	86	110	92	day5
84	81	88				
75	63	86				
57	59	60				
37	39	34				
28	26	23				
19	18	21				
14	14	15				
106	99	102	92	96	105	
84	90	91				
73	62	77				
42	43	51				
23	25	27				
16	20	18				
13	10	13				
8	10	10				
