# Supplementary material for: A novel epigenetic modulating agent sensitizes pancreatic cells to a chemotherapy agent
Source: PLoS One. 2018 Jun 21;13(6):e0199130. doi: 10.1371/journal.pone.0199130 (PMC6013229; doi:10.1371/journal.pone.0199130)
Supplement: S1 File — The archive is organized by cell line, with one folder for each cell line. Within each folder, there is one file for each plot in each figure included in the text. The files are named according to the plot names in each panel of each figure, following the convention “”. Each PDF file contains the raw data for the plot that the filename refers to. (ZIP) [file pone.0199130.s001.zip › Supplemental Data File/Miapaca-2/Figure 1b Low dose.pdf]

Figure 1b low dose

0	97	97	103	99	103	101	day1
0.02	103	95	103	106	89	82	
0.04	109	80	86	94	104	88	
0.07	92	88	95	91	80	84	
0.14	84	75	76	94	92	82	
0.29	81	84	82	97	87	89	
0.43	82	88	70	92	86	83	
0.57	72	74	74	101	84	99	
	90	96	105	104	101	104	day2
	86	100	104	99	108	93	
	95	91	93	89	96	83	
	92	105	89	98	100	88	
	98	87	95	108	95	97	
	74	73	90	103	95	80	
	83	97	91	84	95	84	
	72	100	79	92	81	97	
	91	101	109	110	90	98	day3
	75	75	84	84	95	87	
	85	78	83	94	86	79	
	80	74	84	86	88	76	
	73	82	84	97	95	91	
	79	69	79	85	78	85	
	59	67	77	79	74	71	
	62	75	81	86	88	79	
	100	104	95	97	103	102	day4
	88	85	89	96	96	81	
	92	96	89	87	103	97	
	89	88	98	98	92	94	
	69	88	88	84	73	79	
	57	67	65	65	68	60	
	60	48	59	53	55	70	
	46	36	47	53	57	55	
	119	106	97	99	87	92	day5
	72	88	59	73	82	79	
	75	80	70	85	75	76	
	61	66	71	57	71	57	
	66	79	54	66	67	55	
	43	61	44	44	56	47	
	49	38	39	34	59	49	
	67	36	26	39	50	59	
