# Supplementary material for: A novel epigenetic modulating agent sensitizes pancreatic cells to a chemotherapy agent
Source: PLoS One. 2018 Jun 21;13(6):e0199130. doi: 10.1371/journal.pone.0199130 (PMC6013229; doi:10.1371/journal.pone.0199130)
Supplement: S1 File — The archive is organized by cell line, with one folder for each cell line. Within each folder, there is one file for each plot in each figure included in the text. The files are named according to the plot names in each panel of each figure, following the convention “”. Each PDF file contains the raw data for the plot that the filename refers to. (ZIP) [file pone.0199130.s001.zip › Supplemental Data File/Miapaca-2/Figure 2c.pdf]

Figure 2c

control 0 days	89	98	113	92	108	100
SGI 0 days	111	109	120	59	87	106
Control 5 days	100	99	101	96	107	97
SGI 5 days	188	188	211	419	408	392
Control 10 days	108	79	113	110	94	96
SGI 10 days	180	166	174	138	152	137
