# Supplementary material for: A novel epigenetic modulating agent sensitizes pancreatic cells to a chemotherapy agent
Source: PLoS One. 2018 Jun 21;13(6):e0199130. doi: 10.1371/journal.pone.0199130 (PMC6013229; doi:10.1371/journal.pone.0199130)
Supplement: S1 File — The archive is organized by cell line, with one folder for each cell line. Within each folder, there is one file for each plot in each figure included in the text. The files are named according to the plot names in each panel of each figure, following the convention “”. Each PDF file contains the raw data for the plot that the filename refers to. (ZIP) [file pone.0199130.s001.zip › Supplemental Data File/Miapaca-2/Figure 2d no rest.pdf]

Figure 2d	Control	SGI
	8.128078	0.246305
	25.7329	14.98371
	13.58381	8.092485
	4.915254	5.932203
	4.440497	9.058615
	-0.646552	3.448276
	5.172414	-3.940887
	-7.491857	11.72638
	-1.734104	4.624278
	2.881356	2.20339
	-0.71048	7.992895
	-1.939655	-8.189655
	6.403941	0.492611
	28.6645	17.91531
	-0.578035	-0.578035
	2.033898	8.305085
	2.841918	-0.17762
	-2.801724	11.85345
		8.866995
		24.10423
		-3.179191
		6.271186
		5.150977
		3.87931
		3.694581
		14.00651
		2.023121
		9.661017
		6.394316
		26.2931
	8.640553	9.331798
	2.777778	7.87037
	-0.160901	7.079646
	-0.191449	5.360561
	-1.328021	5.444887
	-1.609517	6.508048
	4.723502	4.953917
	3.935185	8.873457
	5.872888	8.849558
	2.233567	8.296107
	2.52324	5.577689
	2.869139	6.508048
	6.336406	6.336406
	5.015432	6.404321
	6.11424	5.953339
	5.041481	8.679005

4.183267	7.237716
2.659202	7.627712
	15.20737
	5.709877
	5.872888
	7.657945
	12.8154
	8.117565
	11.17512
	1.697531
	9.090909
	11.23165
	12.5498
	9.377187
