# Supplementary material for: A novel epigenetic modulating agent sensitizes pancreatic cells to a chemotherapy agent
Source: PLoS One. 2018 Jun 21;13(6):e0199130. doi: 10.1371/journal.pone.0199130 (PMC6013229; doi:10.1371/journal.pone.0199130)
Supplement: S1 File — The archive is organized by cell line, with one folder for each cell line. Within each folder, there is one file for each plot in each figure included in the text. The files are named according to the plot names in each panel of each figure, following the convention “”. Each PDF file contains the raw data for the plot that the filename refers to. (ZIP) [file pone.0199130.s001.zip › Supplemental Data File/Miapaca-2/Figure 2d rest.pdf]

Figure 2d	control	SGI
	-0.068259	13.65188
	-0.245851	12.10817
	-1.446655	12.4774
	-0.851789	10.33504
	0	11.25901
	0.860092	12.90138
	0.614335	12.35495
	-0.676091	13.82913
	-1.506932	10.91019
	-0.056786	10.16468
	-1.053799	10.64892
	-0.688073	11.63991
		13.7884
		12.84573
		10.84991
		10.22147
		10.70438
		13.99083
		11.05802
		13.03012
		11.75407
		10.10789
		12.81198
		14.1055
	-0.832342	42.05311
	0.712946	32.53283
		37.09244
		20.5298
		15.99198
		17.30696
		22.07689
		18.27392
		18.60376
		18.64761
		23.68737
		-4.146063
		26.75386
		16.02251
		20.98197
		18.36877
		23.68737
		-4.146063
		17.79627
		17.37336
		20.52167
		7.981875

	12.54509
	14.26398
	-1.62505
	24.57786
	22.5163
	9.376089
	18.07615
	4.526436
-3.416436	24.37673
0.470367	22.9539
-2.55102	23.06122
0.959488	29.10448
4.230769	42.05128
13.91905	42.15202
-2.770083	19.66759
0.56444	23.61242
-1.836735	22.04082
1.279318	27.93177
3.205128	-10.64103
17.1767	33.7611
-3.047091	18.00554
-1.881468	20.60207
-1.734694	24.18367
1.279318	26.33262
2.820513	32.30769
17.07799	40.07897
	21.2373
	19.00282
	-6.122449
	16.84435
	23.84615
	33.36624
	22.62234
	22.10724
	24.08163
	27.39872
	23.33333
	43.23791
