# Supplementary material for: A novel epigenetic modulating agent sensitizes pancreatic cells to a chemotherapy agent
Source: PLoS One. 2018 Jun 21;13(6):e0199130. doi: 10.1371/journal.pone.0199130 (PMC6013229; doi:10.1371/journal.pone.0199130)
Supplement: S1 File — The archive is organized by cell line, with one folder for each cell line. Within each folder, there is one file for each plot in each figure included in the text. The files are named according to the plot names in each panel of each figure, following the convention “”. Each PDF file contains the raw data for the plot that the filename refers to. (ZIP) [file pone.0199130.s001.zip › Supplemental Data File/Miapaca-2/Figure 3a no rest.pdf]

Figure 3a		Control											
	0	102	98	102	100	108	105	93	93	93	98	104	103
	0.5	100	97	106	103	108	102						
	1	104	99	114	102	109	103						
	2	91	98	102	102	100	97						
	3	65	67	72	71	72	70						
	4	46	47	43	45	43	46						
		Control											
		99	105	102	106	103	99	90	95	100	101	99	99
		96	104	101	101	99	101						
		92	95	102	106	128	97						
		96	95	100	101	100	103						
		86	84	95	93	95	90						
		66	61	68	71	72	72						
		SGI											
		97	99	102	108	102	103	80	98	102	100	105	103
		95	93	102	99	100	99						
		90	94	94	93	103	93						
		73	74	83	77	78	83						
		40	43	52	48	54	50						
		26	27	34	34	38	36						
		SGI											
		93	103	103	100	100	97	91	103	108	103	98	102
		97	101	101	100	104	98						
		95	115	103	108	105	102						
		89	88	92	95	92	92						
		69	71	77	76	75	79						
		48	55	59	60	59	51						
