# Supplementary material for: A novel epigenetic modulating agent sensitizes pancreatic cells to a chemotherapy agent
Source: PLoS One. 2018 Jun 21;13(6):e0199130. doi: 10.1371/journal.pone.0199130 (PMC6013229; doi:10.1371/journal.pone.0199130)
Supplement: S1 File — The archive is organized by cell line, with one folder for each cell line. Within each folder, there is one file for each plot in each figure included in the text. The files are named according to the plot names in each panel of each figure, following the convention “”. Each PDF file contains the raw data for the plot that the filename refers to. (ZIP) [file pone.0199130.s001.zip › Supplemental Data File/Miapaca-2/Figure 3a rest.pdf]

Figure 3a	Control								
0	94	97	100	101	92	110	99	102	105
0.5	111	116							
1	107	110	113						
2	99	107	90						
3	70	63	75	74	62	78			
4	43	41	47	43	41	37			
	Control								
	96	106	104	101	92	101			
	99	97	89						
	94	92	84						
	105	93	94						
	101	111	100						
	101	92	97						
	SGI								
	97	106	92	101	99	106	94	101	104
	95	104	101						
	93	104	96						
	74	89	84						
	50	50	53	50	47	55			
	32	31	29	35	33	38			
	SGI								
	107	104	107	88	93	99			
	93	90	96						
	88	78	94						
	77	78	82						
	70	61	59						
	45	48	47						
