# Supplementary material for: A novel epigenetic modulating agent sensitizes pancreatic cells to a chemotherapy agent
Source: PLoS One. 2018 Jun 21;13(6):e0199130. doi: 10.1371/journal.pone.0199130 (PMC6013229; doi:10.1371/journal.pone.0199130)
Supplement: S1 File — The archive is organized by cell line, with one folder for each cell line. Within each folder, there is one file for each plot in each figure included in the text. The files are named according to the plot names in each panel of each figure, following the convention “”. Each PDF file contains the raw data for the plot that the filename refers to. (ZIP) [file pone.0199130.s001.zip › Supplemental Data File/Miapaca-2/Figure 4a no rest.pdf]

Figure 4a	Control					
0	9.504132	1.846966	7.325269	2.1777	2.526132	3.222996
0.5	0.068871	-0.263852	-1.075269	2.656794	2.61324	0.391986
1	1.790634	2.37467	3.629032	3.876307	4.355401	3.310105
2	4.820937	6.926121	4.233871	10.67073	9.581882	8.318815
4	15.427	15.56728	12.16398	24.08537	22.95296	20.86237
	SGI					
	7.64045	5.873606	2.566159	5.769931	5.988351	7.571897
	4.794007	4.460967	2.806736	2.493629	5.387696	3.421915
	6.367041	4.832714	4.089816	6.752821	5.551511	10.08373
	11.7603	12.7881	8.981556	14.67055	22.75209	16.09028
	18.8764	21.41264	19.56696	35.03822	42.84674	36.40335
