# Supplementary material for: A novel epigenetic modulating agent sensitizes pancreatic cells to a chemotherapy agent
Source: PLoS One. 2018 Jun 21;13(6):e0199130. doi: 10.1371/journal.pone.0199130 (PMC6013229; doi:10.1371/journal.pone.0199130)
Supplement: S1 File — The archive is organized by cell line, with one folder for each cell line. Within each folder, there is one file for each plot in each figure included in the text. The files are named according to the plot names in each panel of each figure, following the convention “”. Each PDF file contains the raw data for the plot that the filename refers to. (ZIP) [file pone.0199130.s001.zip › Supplemental Data File/Miapaca-2/Figure 4a rest.pdf]

Figure 4a	Control					
0	15.32365	2.38837	7.088608	0.492438	0.318021	1.290998
0.5	10.30383	3.322949	4.219409	2.919451	1.201413	1.953943
1	1.453104	-4.776739	4.556962	3.341541	2.720848	3.942777
2	15.19155	12.04569	16.37131	9.813578	9.222615	8.827634
4	48.61295	24.71443	15.61181	15.16004	15.5477	16.64341
	SGI					
	1.331854	-3.428971	-2.300885	1.91458	1.134142	2.052672
	0	-3.358992	0.973451	5.228277	4.380133	1.239349
	-0.665927	-4.96851	3.185841	7.216495	7.547908	5.344694
	12.20866	5.248425	14.77876	12.96024	12.59288	12.08366
	22.64151	14.3457	16.1062	19.18262	14.43097	14.17506
