# Supplementary material for: A novel epigenetic modulating agent sensitizes pancreatic cells to a chemotherapy agent
Source: PLoS One. 2018 Jun 21;13(6):e0199130. doi: 10.1371/journal.pone.0199130 (PMC6013229; doi:10.1371/journal.pone.0199130)
Supplement: S1 File — The archive is organized by cell line, with one folder for each cell line. Within each folder, there is one file for each plot in each figure included in the text. The files are named according to the plot names in each panel of each figure, following the convention “”. Each PDF file contains the raw data for the plot that the filename refers to. (ZIP) [file pone.0199130.s001.zip › Supplemental Data File/Miapaca-2/Figure 5a no rest.pdf]

Figure 5a		control					
	0	100	99	101	96	107	97
	0.5	130	123	138	125	93	75
	1	149	152	180	114	79	79
	2	174	174	181	149	150	134
		Sgi					
		188	188	211	419	408	392
		191	182	202	219	216	214
		200	215		212	233	227
		156	155	168	230	249	238
