# Supplementary material for: A novel epigenetic modulating agent sensitizes pancreatic cells to a chemotherapy agent
Source: PLoS One. 2018 Jun 21;13(6):e0199130. doi: 10.1371/journal.pone.0199130 (PMC6013229; doi:10.1371/journal.pone.0199130)
Supplement: S1 File — The archive is organized by cell line, with one folder for each cell line. Within each folder, there is one file for each plot in each figure included in the text. The files are named according to the plot names in each panel of each figure, following the convention “”. Each PDF file contains the raw data for the plot that the filename refers to. (ZIP) [file pone.0199130.s001.zip › Supplemental Data File/Miapaca-2/Figure 5a rest.pdf]

Figure 5a

0	108	79	113	110	94	96	Control
0.5	127	122	124	106	113	114	
1	184	175	184	139	87	116	
2	236	233	247	110	98	93	
	180	166	174	138	152	137	SGI
	195	198	178	173	187	146	
	243	253	241	179	170	157	
	301	292	282	204	191	202	
