# Supplementary material for: A novel epigenetic modulating agent sensitizes pancreatic cells to a chemotherapy agent
Source: PLoS One. 2018 Jun 21;13(6):e0199130. doi: 10.1371/journal.pone.0199130 (PMC6013229; doi:10.1371/journal.pone.0199130)
Supplement: S1 File — The archive is organized by cell line, with one folder for each cell line. Within each folder, there is one file for each plot in each figure included in the text. The files are named according to the plot names in each panel of each figure, following the convention “”. Each PDF file contains the raw data for the plot that the filename refers to. (ZIP) [file pone.0199130.s001.zip › Supplemental Data File/Panc1/Figure 1a.pdf]

Figure 1a	Aza							
	0	96	106	101	89	95	113	Day 1
	0.25	94	102	100				
	0.5	95	96	106				
	1	89	102	109				
	2	97	91	101				
	3	94	95	90				
	4	93	93	88				
	5	96	87	84				
		123	103	79	100	105	90	
		107	105	98				
		91	112	86				
		81	81	77				
		67	99	92				
		97	99	114				
		59	57	64				
		70	62	65				
		99	116	102	89	99	95	
		77	95	95				
		78	99	104				
		85	94	88				
		89	92	87				
		85	88	102				
		78	92	89				
		104	106	94	88	101	107	Day 2
		96	109	101				
		92	101	110				
		105	93	106				
		97	99	107				
		96	101	100				
		93	87	85				
		94	88	84				
		113	112	111	65	93	106	
		80	81	109				
		76	120	111				
		76	98	110				
		82	94	101				
		84	94	94				
		47	88	81				
		54	55	60				
		92	93	91	112	104	109	

96	92	88				
84	102	90				
86	101	99				
95	97	97				
93	96	94				
91	92	91				
91	95	96	111	100	106	Day 3
98	104	100				
90	113	111				
88	102	104				
83	95	83				
75	67	63				
59	55	53				
53	47	48				
95	98	99	89	110	110	
104	105	90				
106	108	85				
95	102	92				
76	87	86				
82	90	78				
53	51	50				
43	39	45				
95	107	99	102	96	100	
90	73	100				
99	100	106				
95	76	70				
83	84	112				
87	82	78				
82	68	67				
103	98	105	98	95	101	Day 4
102	111	98				
98	92	99				
100	95	86				
85	76	72				
55	46	44				
41	35	38				
38	32	33				
107	123	132	86	73	78	
122	123	129				
117	103	119				

113	110	116				
90	93	90				
77	65	66				
48	45	41				
28	25	20				
99	107	103	99	101	92	
99	102	99				
97	106	99				
96	100	92				
100	96	92				
53	43	45				
35	32	33				
98	104	100	96	97	105	Day 5
101	102	105				
103	108	106				
103	98	101				
72	54	47				
35	25	24				
23	18	21				
21	17	18				
104	101	103	100	97	96	
106	107	113				
107	111	109				
104	112	110				
93	83	92				
68	53	59				
38	34	33				
27	25	26				
