# Supplementary material for: A novel epigenetic modulating agent sensitizes pancreatic cells to a chemotherapy agent
Source: PLoS One. 2018 Jun 21;13(6):e0199130. doi: 10.1371/journal.pone.0199130 (PMC6013229; doi:10.1371/journal.pone.0199130)
Supplement: S1 File — The archive is organized by cell line, with one folder for each cell line. Within each folder, there is one file for each plot in each figure included in the text. The files are named according to the plot names in each panel of each figure, following the convention “”. Each PDF file contains the raw data for the plot that the filename refers to. (ZIP) [file pone.0199130.s001.zip › Supplemental Data File/Panc1/Figure 1d High dose.pdf]

Figure																				
1d	High	Dose																		
	0	101	107	101	98	102	91	88	85	88	115	120	104	99	99	131	88	92	92	day 1
	0.45	98	112	107	84	72	66	107	91	93										
	0.9	102	112	93	100	91	84	119	113	105										
	1.8	100	108	99	82	86	91	101	101	98										
	3.6	94	102	94	82	103	88	95	102	110										
	5.4	104	102	94	112	129	96													
	7.2	99	93	75	81	91	95	99	130	136										
	9	93	93	84	78	101	90	94	114	96										
		101	101	97	101	103	97	115	110	96	96	98	85	93	90	104	108	106	100	day2
		104	110	102	85	83	77	99	98	89										
		109	104	93	111	100	86	103	102	97										
		108	99	110	91	93	64	112	105	109										
		106	95	105	95	92	66	103	109	102										
		105	104	106	107	103	108													
		104	104	98	101	91	86	98	94	102										
		95	95	92	72	78	67	97	93	96										
		98	100	106	98	98	100	93	103	83	115	108	98	113	106	98	95	92	96	day3
		88	87	82	95	100	86	92	87	95										
		78	79	74	91	91	98	105	85	86										
		79	81	79	80	80	69	75	85	84										
		78	79	74	75	80	88	105	85	86										
		70	72	69	79	75	80													
		66	66	64	57	67	70	85	74	78										
		63	63	63	66	68	83	77	74	78										
		98	102	100	93	96	110	100	101	116	94	89	100	110	105	107	96	88	94	day4
		84	83	78	94	86	103	79	73	76										
		66	66	69	69	73	79	60	62	58										
		75	78	70	83	92	82	61	62	65										
		66	66	69	69	73	79	60	62	58										
		60	61	68	58	57	56													
		57	59	65	73	71	66	55	52	55										
		57	56	63	71	71	71	56	54	54										
		103	99	106	98	93	101	104	110	106	92	94	94							day5
		75	70	71	67	67	62													
		56	52	56	53	48	45													
		59	61	67	65	64	60													
		56	52	56	53	48	45													
		51	55	48	47	47	42													
		50	49	47	46	40	40													
		50	47	49	40	41	40													
