# Supplementary material for: A novel epigenetic modulating agent sensitizes pancreatic cells to a chemotherapy agent
Source: PLoS One. 2018 Jun 21;13(6):e0199130. doi: 10.1371/journal.pone.0199130 (PMC6013229; doi:10.1371/journal.pone.0199130)
Supplement: S1 File — The archive is organized by cell line, with one folder for each cell line. Within each folder, there is one file for each plot in each figure included in the text. The files are named according to the plot names in each panel of each figure, following the convention “”. Each PDF file contains the raw data for the plot that the filename refers to. (ZIP) [file pone.0199130.s001.zip › Supplemental Data File/Panc1/Figure 1d Low dose.pdf]

Figure 1d

0	95	90	81	97	76	80	88	88	244	89	82	89	Day 1
0	75	90	89	94	70	72							
0	84	61	118	106	91	96							
0.1	91	75	100	109	127	98							
0.1	90	107	90	97	114	78							
0.3	86	99	87	100	79	78							
0.4	58	97	98	108	110	83							
0.6	105	100	106	106	110	95							
	98	115	112	100	88	118	83	85	100	115	92	95	Day 2
	90	107	105	115	94	94							
	102	106	100	120	102	97							
	104	119	112	112	104	101							
	100	121	112	123	110	107							
	110	120	109	138	117	100							
	90	120	103	127	93	122							
	108	113	110	130	115	122							
	102	112	107	92	100	103	92	87	87	92	115	111	Day 3
	89	97	103	94	102	101							
	111	107	126	107	104	101							
	105	113	104	109	101	110							
	85	103	115	109	107	113							
	79	113	98	119	90	108							
	110	101	77	112	97	119							
	94	103	102	107	102	107							
	95	123	84	100	110	92	79	118	80	110	106	103	Day 4
	71	119	100	95	105	91							
	76	118	84	106	115	100							
	125	107	99	96	111	106							
	99	127	96	143	127	125							
	42	91	94	102	106	96							
	91	85	89	86	92	103							
	83	82	71	80	81	114							
	99	102	106	102	99	100	95	96	101	104	98	99	Day 5
	104	101	98	99	102	96							
	95	96	105	103	104	93							
	90	96	102	105	102	99							
	103	104	106	111	108	103							
	103	103	103	107	105	99							
	99	102	98	100	100	86							
	92	88	93	92	92	94							
