# Supplementary material for: A novel epigenetic modulating agent sensitizes pancreatic cells to a chemotherapy agent
Source: PLoS One. 2018 Jun 21;13(6):e0199130. doi: 10.1371/journal.pone.0199130 (PMC6013229; doi:10.1371/journal.pone.0199130)
Supplement: S1 File — The archive is organized by cell line, with one folder for each cell line. Within each folder, there is one file for each plot in each figure included in the text. The files are named according to the plot names in each panel of each figure, following the convention “”. Each PDF file contains the raw data for the plot that the filename refers to. (ZIP) [file pone.0199130.s001.zip › Supplemental Data File/Panc1/Figure 2a.pdf]

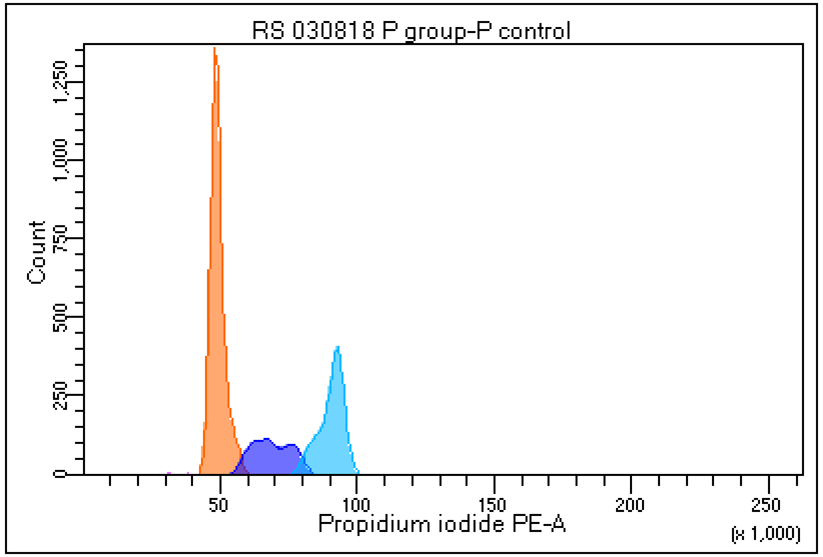

Experiment Name: Cell cycle 030818
Specimen Name: RS 030818 P group
Tube Name: P control
Record Date: Mar 8, 2018 1:26:42 PM
$OP:
GUID: d6f2d986-719c-4669-8cd1...

Population	#Events	%Parent
All Events	21,469	#####
P1	17,649	82.2
P2	13,993	79.3
G1	7,264	51.9
S	2,458	17.6
G2_M	3,886	27.8
Sub G0/G1	162	1.2

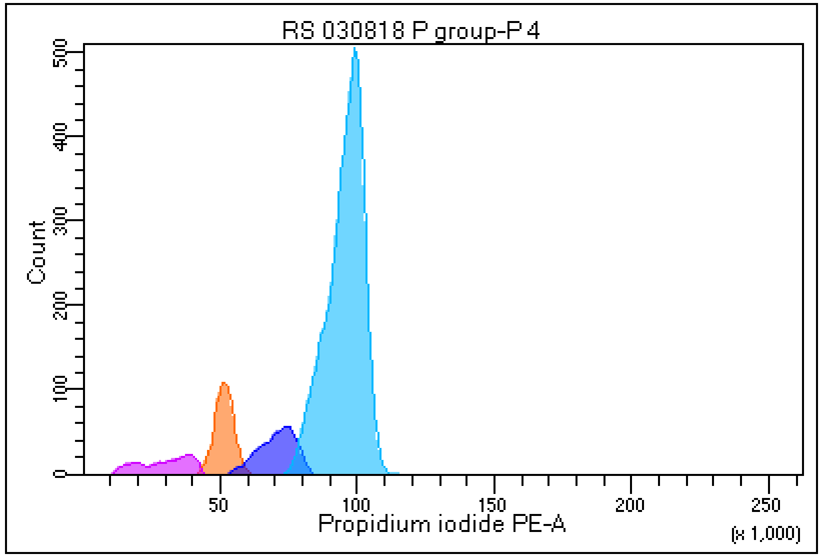

Experiment Name: Cell cycle 030818
Specimen Name: RS 030818 P group
Tube Name: P 4
Record Date: Mar 8, 2018 1:34:49 PM
$OP:
GUID: f8d3ea79-eb1f-45d5-bd14...

Population	#Events	%Parent
All Events	37,401	#####
P1	31,173	83.3
P2	10,071	32.3
G1	981	9.7
S	991	9.8
G2_M	7,114	70.6
Sub G0/G1	570	5.7

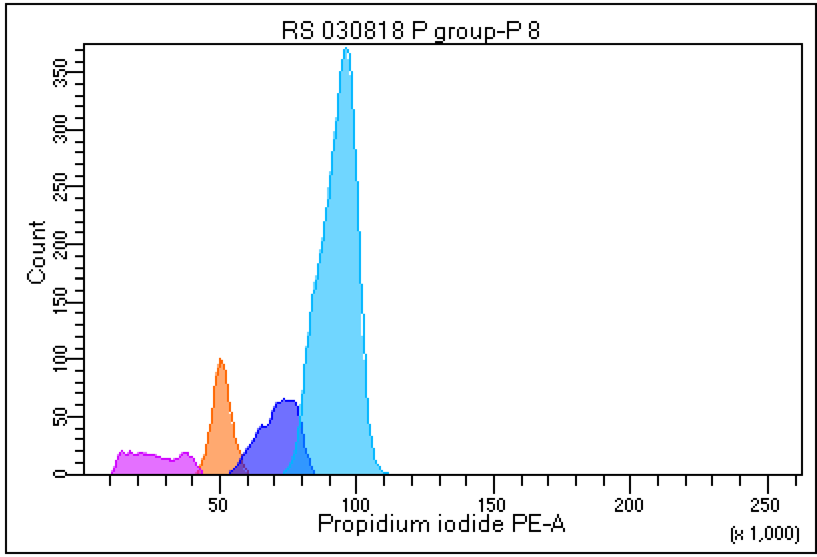

Experiment Name: Cell cycle 030818
Specimen Name: RS 030818 P group
Tube Name: P 8
Record Date: Mar 8, 2018 1:41:22 PM
$OP:
GUID: 5a70e576-d9a1-49c2-ac6...

Population	#Events	%Parent
All Events	32,107	#####
P1	27,326	85.1
P2	8,827	32.3
G1	847	9.6
S	1,200	13.6
G2_M	5,694	64.5
Sub G0/G1	570	6.5

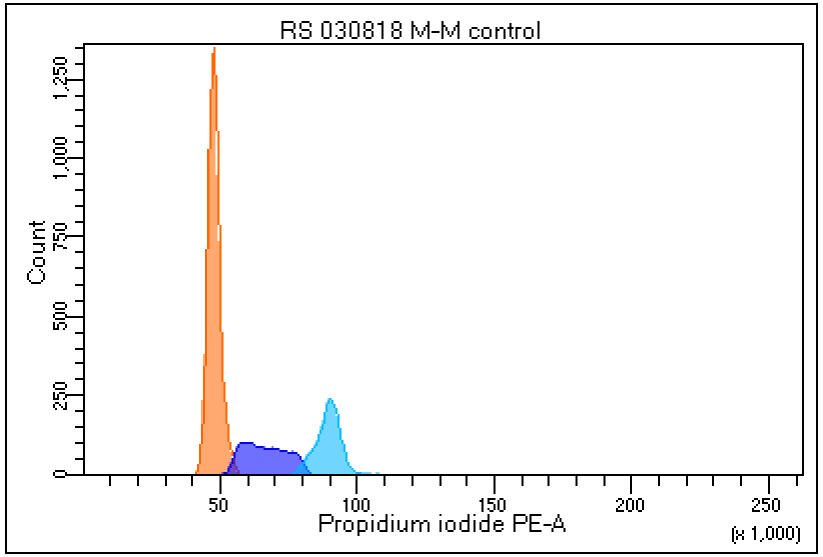

Experiment Name:	Cell cycle 030818
Specimen Name:	RS 030818 M
Tube Name:	M control
Record Date:	Mar 8, 2018 1:43:37 PM
$OP:	
GUID:	0408251d-f330-4b2b-982...

Population	#Events	%Parent
All Events	17,445	#####
P1	16,651	95.4
P2	12,332	74.1
G1	6,858	55.6
S	2,425	19.7
G2_M	2,613	21.2
Sub G0/G1	106	0.9

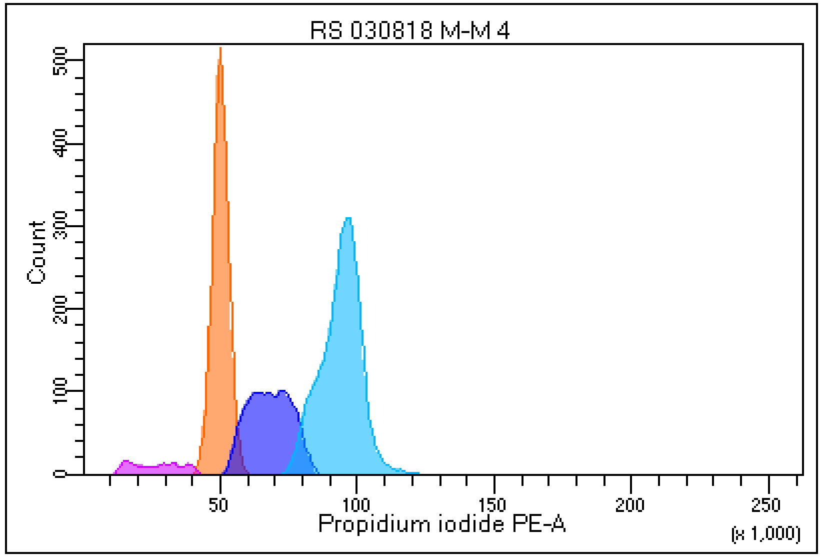

Experiment Name:	Cell cycle 030818
Specimen Name:	RS 030818 M
Tube Name:	M 4
Record Date:	Mar 8, 2018 1:47:27 ...
$OP:	
GUID:	f667cd3d-b465-4d73-...

Population	#Events	%Parent
All Events	26,608	#####
P1	4,746	17.8
P2	2,437	51.3
G1	332	13.6
S	440	18.1
G2_M	1,600	65.7
Sub G0/G1	11	0.5

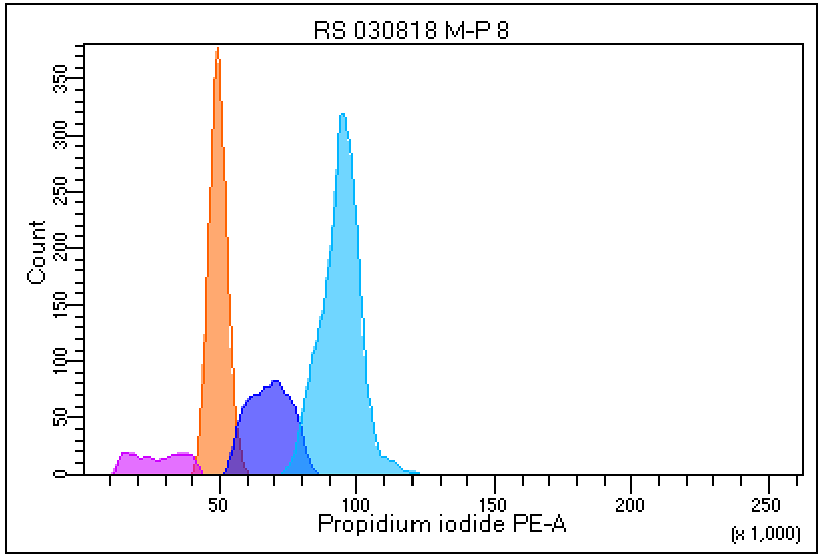

Experiment Name:	Cell cycle 030818
Specimen Name:	RS 030818 M
Tube Name:	P 8
Record Date:	Mar 8, 2018 1:53:47 PM
$OP:	
GUID:	a917380f-66b2-40f4-8c32-...

Population	#Events	%Parent
All Events	27,307	#####
P1	25,425	93.1
P2	10,514	41.4
G1	2,927	27.8
S	1,834	17.4
G2_M	4,904	46.6
Sub G0/G1	555	5.3
