# Supplementary material for: A novel epigenetic modulating agent sensitizes pancreatic cells to a chemotherapy agent
Source: PLoS One. 2018 Jun 21;13(6):e0199130. doi: 10.1371/journal.pone.0199130 (PMC6013229; doi:10.1371/journal.pone.0199130)
Supplement: S1 File — The archive is organized by cell line, with one folder for each cell line. Within each folder, there is one file for each plot in each figure included in the text. The files are named according to the plot names in each panel of each figure, following the convention “”. Each PDF file contains the raw data for the plot that the filename refers to. (ZIP) [file pone.0199130.s001.zip › Supplemental Data File/Panc1/Figure 2c.pdf]

Figure 2c

control 0 days	97	98	105	94	107	99
SGI 0 days	106	85	94	111	113	114
Control 5 days	105	102	93	102	101	98
SGI 5 days	262	248	251	279	285	296
Control 0 days	101	101	98	103	96	101
SGI 10 days	220	181	218	167	163	160
