# Supplementary material for: A novel epigenetic modulating agent sensitizes pancreatic cells to a chemotherapy agent
Source: PLoS One. 2018 Jun 21;13(6):e0199130. doi: 10.1371/journal.pone.0199130 (PMC6013229; doi:10.1371/journal.pone.0199130)
Supplement: S1 File — The archive is organized by cell line, with one folder for each cell line. Within each folder, there is one file for each plot in each figure included in the text. The files are named according to the plot names in each panel of each figure, following the convention “”. Each PDF file contains the raw data for the plot that the filename refers to. (ZIP) [file pone.0199130.s001.zip › Supplemental Data File/Panc1/Figure 2d no rest.pdf]

Figure 2d	Control	SGI
	-51.38889	-43.05556
	34.66667	24.66667
	2.317881	7.284768
	150	316.6667
	-7.692307	-1.923077
	2.027027	6.756757
	-94.44444	-30.55556
	18.66667	42
	5.960265	10.92715
	166.6667	75
	-10.25641	30.76923
	19.59459	31.75676
	-94.44444	-76.38889
	25.33333	28.66667
	13.57616	18.54305
	8.333333	41.66667
	11.53846	8.974359
	16.89189	26.35135
		-37.5
		12
		20.5298
		-8.333333
		22.4359
		25
		11.11111
		29.33333
		21.52318
		-275
		5.128205
		26.35135
	-1.713796	-0.599829
	-2.565632	-0.536993
	-1.550388	3.255814
	1.566872	3.077784
	0.923077	3.333333
	0.390625	3.180804
	-1.028278	-1.199657
	-3.162291	2.02864
	1.627907	3.953488
	2.238388	0.223839
	0.205128	3.948718
	-1.953125	2.734375
	-3.513282	-0.17138
	-2.804296	-0.417661
	4.108527	2.325581
	3.637381	1.175154

1.74359	2.153846
0.111607	1.506696
	-0.599829
	-2.207637
	2.248062
	-2.070509
	-1.282051
	-0.558036
	-1.285347
	-2.446301
	0.387597
	-0.05596
	3.128205
	-2.399554
