# Supplementary material for: A novel epigenetic modulating agent sensitizes pancreatic cells to a chemotherapy agent
Source: PLoS One. 2018 Jun 21;13(6):e0199130. doi: 10.1371/journal.pone.0199130 (PMC6013229; doi:10.1371/journal.pone.0199130)
Supplement: S1 File — The archive is organized by cell line, with one folder for each cell line. Within each folder, there is one file for each plot in each figure included in the text. The files are named according to the plot names in each panel of each figure, following the convention “”. Each PDF file contains the raw data for the plot that the filename refers to. (ZIP) [file pone.0199130.s001.zip › Supplemental Data File/Panc1/Figure 2d rest.pdf]

Figure 2d

	Control	SGI
	-0.439883	9.67742
	0.776288	5.081157
	-3.032544	3.476331
	4.405594	4.685315
	-0.564617	5.583438
	-0.567376	4.680851
	0.513197	4.2522
	-0.211715	4.587156
	-1.035503	3.846154
	-0.34965	11.25874
	-0.188206	6.712673
	0.35461	5.531915
	0.146628	7.917889
	1.340861	6.774877
	-2.071006	5.473373
	-1.538462	9.230769
	-0.062735	6.273526
	-0.141844	5.602837
		7.917889
		10.16231
		4.659763
		6.713287
		7.904643
		7.659575
		7.478006
		10.4446
		10.50296
		7.412588
		7.214555
		5.886525
	5.508474	24.57627
	-6.288533	20.34525
	-1.19403	29.40298
	4.23913	19.45652
	20.04608	37.17358
	-3.608248	18.04124
	-2.436441	25.21186
	3.452528	32.67571
	-0.447761	30.14925
	-2.608696	20.43478
	22.19662	41.62827
	-1.237113	22.37113
		22.45763
		22.19482
		29.10448
		18.69565

35.17665
11.54639
21.61017
19.35882
20.59702
14.34783
36.55914
13.60825
23.09322
18.74229
28.20896
17.5
39.55453
16.49484
