# Supplementary material for: A novel epigenetic modulating agent sensitizes pancreatic cells to a chemotherapy agent
Source: PLoS One. 2018 Jun 21;13(6):e0199130. doi: 10.1371/journal.pone.0199130 (PMC6013229; doi:10.1371/journal.pone.0199130)
Supplement: S1 File — The archive is organized by cell line, with one folder for each cell line. Within each folder, there is one file for each plot in each figure included in the text. The files are named according to the plot names in each panel of each figure, following the convention “”. Each PDF file contains the raw data for the plot that the filename refers to. (ZIP) [file pone.0199130.s001.zip › Supplemental Data File/Panc1/Figure 3b no rest.pdf]

Figure 3b

0	16	128	161	179	144	46	7	93	144	150	114	18	Control
0.5	41	150	181	176	158	57							
1	43	156	200	204	168	60							
2	74	161	197	196	167	61							
4	90	151	164	175	155	64							
	98	96	104	105	105	106	90	97	95	104	101	99	
	94	96	104	104	102	105							
	98	102	103	100	100	107							
	100	105	102	104	103	107							
	92	96	100	98	96	95							
	19	106	127	144	141	115	-8	87	119	129	125	96	SGI
	47	126	132	144	143	114							
	72	123	140	143	130	118							
	63	97	115	115	109	95							
	45	55	53	59	54	52							
	93	101	104	104	100	107	92	96	91	103	101	107	
	82	92	97	98	99	102							
	82	88	99	99	93	86							
	76	74	86	89	84	87							
	49	52	57	57	62	61							
