# Supplementary material for: A novel epigenetic modulating agent sensitizes pancreatic cells to a chemotherapy agent
Source: PLoS One. 2018 Jun 21;13(6):e0199130. doi: 10.1371/journal.pone.0199130 (PMC6013229; doi:10.1371/journal.pone.0199130)
Supplement: S1 File — The archive is organized by cell line, with one folder for each cell line. Within each folder, there is one file for each plot in each figure included in the text. The files are named according to the plot names in each panel of each figure, following the convention “”. Each PDF file contains the raw data for the plot that the filename refers to. (ZIP) [file pone.0199130.s001.zip › Supplemental Data File/Panc1/Figure 3b rest.pdf]

Figure 3b

0	103	103	107	94	99	92	99	98	106	control
0.5	109	111	105							
1	109	113	107							
2	111	118	105							
4	67	63	73	76	74	81				
	101	101	97	93	104	104				
	109	114	109							
	104	109	108							
	101	102	101							
	89	87	86							
	106	93	109	92	98	102	102	99		SGI
	100	102	96							
	105	91	95							
	87	82	81							
	46	49	51	58	53	51				
	109	100	106	99	90	96				
	99	91	101							
	66	62	63							
	37	40	36							
	66	62	63							
