# Supplementary material for: A novel epigenetic modulating agent sensitizes pancreatic cells to a chemotherapy agent
Source: PLoS One. 2018 Jun 21;13(6):e0199130. doi: 10.1371/journal.pone.0199130 (PMC6013229; doi:10.1371/journal.pone.0199130)
Supplement: S1 File — The archive is organized by cell line, with one folder for each cell line. Within each folder, there is one file for each plot in each figure included in the text. The files are named according to the plot names in each panel of each figure, following the convention “”. Each PDF file contains the raw data for the plot that the filename refers to. (ZIP) [file pone.0199130.s001.zip › Supplemental Data File/Panc1/Figure 4b no rest.pdf]

Figure 4b

0	1.245675	1.807229	4.089457	-0.276807	-1.019814	-0.101981	Control
0.5	1.314879	1.314879	1.314879	-1.675408	-0.844988	-0.495338	
1	6.920415	5.689425	5.43131	2.695221	2.170746	1.995921	
2	17.37024	13.38688	12.07668	8.071095	8.289627	6.759907	
4	24.49827	23.22624	27.02875	27.12704	25.4662	24.94172	
	10.84259	9.615385	9.299098	0.947274	0.786417	-5.004468	SGI
	1.314879	1.314879	1.314879	-0.768543	-0.661305	-2.484361	
	11.95297	9.350133	8.188758	5.826631	4.057194	3.628239	
	14.23906	18.76658	13.87925	12.85076	14.084	14.78105	
	26.32266	30.70292	27.6891	43.14566	37.89098	40.7328	
