# Supplementary material for: A novel epigenetic modulating agent sensitizes pancreatic cells to a chemotherapy agent
Source: PLoS One. 2018 Jun 21;13(6):e0199130. doi: 10.1371/journal.pone.0199130 (PMC6013229; doi:10.1371/journal.pone.0199130)
Supplement: S1 File — The archive is organized by cell line, with one folder for each cell line. Within each folder, there is one file for each plot in each figure included in the text. The files are named according to the plot names in each panel of each figure, following the convention “”. Each PDF file contains the raw data for the plot that the filename refers to. (ZIP) [file pone.0199130.s001.zip › Supplemental Data File/Panc1/Figure 4b rest.pdf]

Figure 4b

0	22.25859	20.89125	28.50819	-1.571063	-2.444528	-0.448263	Control
0.5	23.07692	1.314879	1.314879	1.315309	1.314879	1.314879	
1	31.34206	29.55846	31.73971	1.351845	1.316284	1.830407	
2	34.57447	32.46116	34.74989	5.590062	4.437758	6.948076	
4	43.3306	45.70728	48.07437	12.78772	10.94396	13.59731	
	34.39673	34.56147	33.97169	-1.63728	-2.349423	-1.505017	SGI
	1.314879	1.314879	1.314879	1.314879	1.314879	1.314879	
	39.0593	39.04686	41.21565	3.232578	1.580521	1.295987	
	40.0409	41.97036	42.21482	4.65995	4.314395	4.09699	
	54.39673	55.6668	61.49043	6.507137	4.399829	2.257525	
