# Supplementary material for: A novel epigenetic modulating agent sensitizes pancreatic cells to a chemotherapy agent
Source: PLoS One. 2018 Jun 21;13(6):e0199130. doi: 10.1371/journal.pone.0199130 (PMC6013229; doi:10.1371/journal.pone.0199130)
Supplement: S1 File — The archive is organized by cell line, with one folder for each cell line. Within each folder, there is one file for each plot in each figure included in the text. The files are named according to the plot names in each panel of each figure, following the convention “”. Each PDF file contains the raw data for the plot that the filename refers to. (ZIP) [file pone.0199130.s001.zip › Supplemental Data File/Panc1/figure 5b no rest.pdf]

Figure 5b

0	105	102	93	102	101	98	Control
0.5	107	101	97	128	123	119	
1	118	116	103	138	140	139	
2	110	83	72	210	206	211	
	262	248	251	279	285	296	SGI
	296	254	278	228	228	201	
	306	278	290	238	229	240	
	372	293	348	297	308	315	
