# Supplementary material for: A novel epigenetic modulating agent sensitizes pancreatic cells to a chemotherapy agent
Source: PLoS One. 2018 Jun 21;13(6):e0199130. doi: 10.1371/journal.pone.0199130 (PMC6013229; doi:10.1371/journal.pone.0199130)
Supplement: S1 File — The archive is organized by cell line, with one folder for each cell line. Within each folder, there is one file for each plot in each figure included in the text. The files are named according to the plot names in each panel of each figure, following the convention “”. Each PDF file contains the raw data for the plot that the filename refers to. (ZIP) [file pone.0199130.s001.zip › Supplemental Data File/Panc1/Figure 5b rest.pdf]

Figure 5b

| | | | | | | | |
|---|---|---|---|---|---|---|---|
| 0 | 101 | 101 | 98 | 103 | 96 | 101 | Control |
| 0.5 | 103 | 105 | 107 | 109 | 113 | 100 | |
| 1 | 145 | 126 | 128 | 130 | 146 | 136 | |
| 2 | 269 | 233 | 216 | 195 | 198 | 208 | |
| | | | | | | | |
| | 220 | 181 | 218 | 167 | 163 | 160 | SGI |
| | 263 | 234 | 235 | 173 | 181 | 186 | |
| | 321 | 343 | 332 | 225 | 226 | 236 | |
| | 415 | 435 | 416 | 259 | 269 | 264 | |
