# Supplementary material for: A novel epigenetic modulating agent sensitizes pancreatic cells to a chemotherapy agent
Source: PLoS One. 2018 Jun 21;13(6):e0199130. doi: 10.1371/journal.pone.0199130 (PMC6013229; doi:10.1371/journal.pone.0199130)
Supplement: S1 File — The archive is organized by cell line, with one folder for each cell line. Within each folder, there is one file for each plot in each figure included in the text. The files are named according to the plot names in each panel of each figure, following the convention “”. Each PDF file contains the raw data for the plot that the filename refers to. (ZIP) [file pone.0199130.s001.zip › Supplemental Data File/PL45/Figure 1a.pdf]

Figure 1a	AZA	Day1					
	0	95	107	103	110	94	90
	0.25	92	111	99			
	0.5	93	105	95			
	1	86	76	96			
	2	66	93	98			
	3	66	89	81			
	4	75	78	81			
	5	78	83	78			
		99	106	105	78	107	105
		95	119	126			
		110	118	130			
		112	92	111			
		110	120	119			
		88	121	114			
		78	95	104			
		78	96	75			
		84	112	112	93	89	111
		107	105	100			
		96	118	95			
		85	100	114			
		84	105	101			
		89	90	111			
		87	94	108			
		74	70	99			
		Day 2					
		101	94	96	101	107	101
		105	108	83			
		113	109	118			
		99	104	99			
		100	102	91			
		87	97	86			
		76	79	80			
		78	66	78			
		87	108	104	101	102	99
		101	82	96			
		95	96	97			
		96	95	98			
		82	77	71			
		70	65	74			
		54	55	49			
		51	49	51			

		103	94	92	99	108	104
		101	85	87			
		100	100	89			
		98	92	98			
		82	82	80			
		72	77	78			
		73	69	82			
		52	57	55			
Day 3		100	70	97	118	115	101
		43	71	87			
		95	77	78			
		96	84	90			
		86	87	88			
		66	72	75			
		65	68	63			
		54	64	67			
		102	104	100	88	98	107
		95	99	105			
		102	105	106			
		86	95	104			
		65	65	65			
		47	46	50			
		28	35	37			
		10	24	23			
		99	103	104	96	108	90
		99	94	95			
		92	92	97			
		86	93	92			
		76	78	78			
		64	70	67			
		61	63	55			
		38	39	39			
	Day 4						
		106	102	96	100	98	100
		86	86	86			
		87	76	87			
		78	82	75			
		64	66	61			
		47	54	55			
		46	49	45			
		46	48	50			
		102	86	55	100	128	129

	74	61	61			
	85	69	49			
	79	74	73			
	65	62	61			
	56	58	62			
	46	49	45			
	57	65	68			
	58	100	52	118	140	132
	110	131	131			
	104	79	45			
	98	83	87			
	82	78	78			
	65	65	62			
	57	50	50			
	31	33	35			
day 5	127	124	122	88	38	102
	125	85	85			
	118	91	77			
	95	78	67			
	75	62	58			
	52	56	50			
	37	37	35			
	25	35	31			
	99	96	75	106	110	113
	92	27	27			
	79	82	68			
	73	85	78			
	58	64	53			
	49	54	52			
	42	45	45			
	55	56	56			
	84	89	82	113	116	116
	86	120	120			
	81	76	71			
	65	45	65			
	48	41	48			
	46	39	37			
	41	29	22			
	27	27	22			
