# Supplementary material for: A novel epigenetic modulating agent sensitizes pancreatic cells to a chemotherapy agent
Source: PLoS One. 2018 Jun 21;13(6):e0199130. doi: 10.1371/journal.pone.0199130 (PMC6013229; doi:10.1371/journal.pone.0199130)
Supplement: S1 File — The archive is organized by cell line, with one folder for each cell line. Within each folder, there is one file for each plot in each figure included in the text. The files are named according to the plot names in each panel of each figure, following the convention “”. Each PDF file contains the raw data for the plot that the filename refers to. (ZIP) [file pone.0199130.s001.zip › Supplemental Data File/PL45/Figure 1c High dose.pdf]

Figure 1c	SGI	High Dose						
	0	103	102	97	99	91	108	day1
	0.45	105	95	87				
	0.9	85	91	93				
	1.8	94	88	83				
	3.6	106	98	89				
	5.4	91	90	82				
	7.2	89	95	87				
	9	88	93	84				
		91	93	99	115	83	120	
		107	114	117				
		129	111	114				
		115	113	119				
		115	117	117				
		114	120	114				
		128	119	123				
		122	128	119				
		107	115	104	94	89	92	
		99	110	108				
		82	93	103				
		90	92	95				
		98	99	100				
		103	97	98				
		99	97	29				
		97	93	96				
		92	98	100	110	104	96	day2
		95	102	88				
		98	101	95				
		94	94	82				
		96	86	86				
		101	84	90				
		97	99	93				
		99	90	103				
		97	101	95	103	102	102	
		99	93	99				
		88	93	103				
		96	99	102				
		96	96	95				
		102	98	95				
		95	99	96				
		99	103	111				
		86	81	71	120	123	119	

95	81	66				
95	82	91				
113	96	102				
119	112	112				
114	117	113				
117	108	116				
112	110	108				
83	93	87	107	111	118	day3
68	79	83				
75	73	76				
79	71	84				
88	83	85				
84	88	87				
90	80	83				
84	86	91				
95	90	86	107	100	122	
82	86	90				
92	87	98				
97	91	85				
88	84	105				
89	92	91				
78	92	92				
83	89	66				
56	117	116	110	105	97	
103	110	109				
100	100	97				
94	98	101				
97	101	106				
94	98	101				
97	100	107				
96	85	96				
108	118	64	105	99	106	day4
72	58	56				
63	67	64				
68	69	64				
63	67	64				
69	67	70				
66	69	64				
68	71	67				
75	79	78	121	123	123	
49	54	70				
66	63	70				

49	56	61			
66	63	70			
75	77	76			
81	81	79			
80	74	78			
64	71	97	121	121	126
50	34	68			
61	68	74			
59	27	69			
61	68	74			
66	67	74			
76	75	68			
74	70	50			
86	86	73	112	122	121 day5
24	40	42			
33	40	36			
28	40	46			
33	40	36			
33	40	39			
37	42	34			
27	49	35			
75	91	90	117	112	115
66	64	69			
44	66	41			
68	66	46			
44	66	41			
53	47	66			
63	65	62			
65	67	65			
88	66	79	131	133	103
57	59	67			
47	57	60			
42	56	61			
47	57	60			
59	54	60			
44	51	50			
50	51	40			
