# Supplementary material for: A novel epigenetic modulating agent sensitizes pancreatic cells to a chemotherapy agent
Source: PLoS One. 2018 Jun 21;13(6):e0199130. doi: 10.1371/journal.pone.0199130 (PMC6013229; doi:10.1371/journal.pone.0199130)
Supplement: S1 File — The archive is organized by cell line, with one folder for each cell line. Within each folder, there is one file for each plot in each figure included in the text. The files are named according to the plot names in each panel of each figure, following the convention “”. Each PDF file contains the raw data for the plot that the filename refers to. (ZIP) [file pone.0199130.s001.zip › Supplemental Data File/PL45/Figure 1c Low dose.pdf]

Figure	SGI												
1c	low	day1											
	0	97.38	90.938	97	100.41	91.317	92.075	109.9	111.8	113.7	99.3	96.243	100
	0.02	92.45	103.06	99.274	95.106	96.243	93.969						
	0.04	99.65	101.93	96.621	89.422	92.832	95.106						
	0.07	95.11	96.621	100.41	97.758	94.348	90.938						
	0.14	97.38	103.06	107.99	102.68	96.243	96.621						
	0.29	105.7	105.72	108.75	101.55	100.79	87.528						
	0.43	111.8	117.46	111.02	84.875	100.79	93.969						
	0.57	112.2	115.19	111.78	90.559	104.2	93.59						
		100	102.95	100.05	102.37	95.116	91.634	96.86	100.3	100.9	106	106.14	97.73
		100	112.81	105.56	106.14	95.696	91.925						
		100.9	104.98	100.92	106.72	102.95	86.992						
		100.6	102.08	109.04	111.94	107.3	89.313						
		104.7	108.46	111.94	119.49	111.94	93.085						
		101.8	108.17	111.36	116.59	107.3	93.665						
		98.02	112.81	109.91	117.46	107.3	93.665						
		101.2	107.59	104.11	108.75	109.04	94.246						
		day 2											
		116	109.28	119.12	117.72	110.69	117.72	51.3	57.98	96.28	94.9	103.66	105.4
		104	108.93	109.63	115.26	108.23	119.12						
		93.12	79.414	103.31	104.71	107.53	110.34						
		56.57	82.577	94.876	108.58	103.66	105.42						
		50.25	68.873	100.85	107.53	106.82	105.07						
		62.2	60.088	82.928	96.633	102.96	105.07						
		48.49	78.009	92.767	100.15	102.26	102.96						
		75.2	63.953	81.171	92.064	94.876	97.335						
		95.54	98.394	100.47	96.836	102.55	97.875	106.4	102	106.7	98.4	94.758	
		101	106.97	96.057	103.85	101.51	98.394						
		94.5	104.37	106.19	101.77	101.77	101.77						
		97.36	96.576	100.73	94.758	108.52	99.174						
		93.72	92.161	104.11	102.29	105.15	103.59						
		99.17	97.355	104.89	106.19	107.23	98.914						
		97.1	100.21	101.51	98.394	97.355	85.668						
		97.62	99.693	102.81	98.135	95.797	96.057						
		day 3											
		103.2	106.94	99.054	104.62	101.14	104.62	95.11	105.8	90.48	96.3	97.431	95.35
		94.88	89.318	93.49	86.537	91.404	95.577						
		91.64	92.1	96.736	88.623	90.477	84.45						
		85.38	86.073	91.868	93.49	89.782	87						
		83.76	88.159	93.722	95.113	90.477	86.537						
		80.28	87.232	88.623	90.477	86.537	86.537						
		85.15	83.06	87.927	88.159	87.464	83.987						

86.07	86.073	87	83.291	84.682	80.742

101.3	108.71	103.29	101.34	99.603	100.25	105	102.9	92.88	91.1	96.351	97.22
98.74	98.085	100.25	93.316	92.666	92.883						
88.11	94.4	99.603	91.366	90.932	95.267						
92.23	89.198	93.1	94.184	88.114	92.883						
86.16	93.75	91.149	92.449	89.198	91.799						
95.27	85.73	90.715	91.799	88.331	87.247						
80.53	89.632	97.868	88.548	88.548	87.464						
86.6	89.198	86.163	87.247	82.262	82.262						

day 4

97.79	95.349	99.114	98.671	98.228	100	103.8	98.89	100	101	102.44	104.7
87.6	88.04	84.053	93.577	84.939	85.825						
86.93	87.154	88.704	85.604	88.261	92.027						
80.73	86.489	83.832	82.946	83.167	85.161						
78.52	79.623	76.966	81.174	76.966	80.288						
78.52	73.2	74.308	75.415	72.979	72.315						
74.31	71.65	71.65	71.429	72.093	70.1						
68.99	65.006	66.556	64.784	62.791	67.22						

93.44	95.618	103.13	100.56	94.827	95.025	102.7	106.1	111	101	96.409	100.6
84.75	90.28	92.85	90.082	86.524	86.722						
86.33	84.547	91.071	86.722	85.931	85.338						
83.56	82.175	86.326	87.908	80.198	77.825						
76.44	77.43	86.722	81.582	77.035	82.768						
72.88	76.639	78.221	78.221	78.616	75.255						
70.51	75.651	76.837	75.651	64.975	68.731						
65.57	73.278	73.871	67.348	66.755	64.58						

day 5

97.02	99.887	103.94	98.874	98.367	101.24	97.19	97.19	97.86	103	98.029	107.3
82.32	82.995	85.698	81.813	82.489	84.009						
84.35	83.84	85.36	83.164	82.827	80.968						
78.1	76.07	82.827	80.462	78.604	78.435						
71.85	70.664	78.941	71.847	72.016	75.901						
63.74	55.968	68.468	62.05	61.543	66.441						
66.1	64.245	67.117	69.144	62.05	66.779						
52.76	57.658	55.124	56.644	55.293	54.786						

100.8	90.702	93.23	95.758	98.792	98.624	106.4	109.2	105	102	97.781	102
83.29	85.309	86.826	84.466	83.455	84.635						
75.7	79.916	81.77	78.904	83.455	79.916						
75.2	73.68	73.174	71.489	77.388	74.522						
70.48	71.489	69.803	69.635	68.118	71.489						
63.74	63.399	64.747	62.893	63.567	64.073						
61.88	63.062	61.545	67.444	57.837	58.511						

57.5	61.039	58.68	56.32	55.478	52.949
